# Examining the relationship between income and both mental and physical health among adults in the UK: Analysis of 12 waves (2009–2022) of Understanding Society

**DOI:** 10.1371/journal.pone.0316792

**Published:** 2025-03-06

**Authors:** Howard Robert Reed, Daniel Nettle, Fiorella Parra-Mujica, Graham Stark, Richard Wilkinson, Matthew Thomas Johnson, Elliott Aidan Johnson

**Affiliations:** 1 Social Work, Education and Community Wellbeing, Northumbria University, Newcastle upon Tyne, United Kingdom; 2 Landman Economics, Colchester, United Kingdom; 3 Institut Jean Nicod, École normale supérieure, Paris, France; 4 School of Health Policy and Management, Erasmus University Rotterdam, Rotterdam, Netherlands; University Putra Malaysia: Universiti Putra Malaysia, MALAYSIA

## Abstract

There is growing evidence of a causal relationship between income and health. At the same time, pressure on reactive health and care services in the UK is increasing. Previous work to quantify the relationship has focused on particular age groups, conditions, or single-item self-rated health. This article reports findings from a study that aimed to provide more comprehensive estimates with an objective of creating an evidential basis for microsimulation modelling of upstream income interventions. We analyse the relationship between income and two health measures – SF-12 Mental Component Summary (MCS-12) and Physical Component Summary (PCS-12) – across 12 waves (2009/11–2020/22) of Understanding Society: The UK Household Longitudinal Study. Using a ‘within-between’ model (Model 1), we find that increases in income compared with an individual’s average and a higher income compared with the sample average is associated with better mental health (higher MCS-12 score) and better functional physical health (higher PCS-12 score). However, for a given increase in household income (say £100 per month), the association with better mental and physical health is smaller at higher incomes. This suggests that redistribution from high-income to lower income households would increase average population physical and mental health, other things being equal. Using a random-effects logistic regression (Model 2), we similarly find that average income quintile is inversely and monotonically associated with the probability of having clinically significant symptoms of depressive disorders (MCS-12 ≤ 45.6) and physical health problems (PCS-12 score ≤ 50.0), with smaller changes in these probabilities from increases in income at higher points in the income distribution. These findings facilitate microsimulation modelling including an estimation of the impact of changes in QALYs, from changes in income, enabling a more detailed and complete understanding of which socioeconomic interventions might begin to address some of the causes of long-term health conditions that are underpinned by socioeconomic determinants.

## Introduction

There is growing consensus on the importance of addressing social determinants as a pillar of public health policy. In the UK, the Government’s Levelling Up White Paper highlighted differences of up to 18 years lived in good health between the most and least deprived areas of Britain [1]. The report added to a body of evidence that income is a key social determinant of health, suggesting that interventions to increase income have potential to improve health at a time of crisis in reactive health services. In prior work, we have suggested that Basic Income offers means of promoting health by reducing poverty and thereby improving the capacity of individuals to satisfy their basic needs, mitigating inequality and thereby reducing stress and enhancing economic security thereby promoting long-term health promoting behaviour [2]. There is evidence of prospective health benefit from upstream financial interventions [3,4] and support for such policies among the public [5]. In the absence of funding for large scale randomised controlled trials [6], policymakers have urgent need to assess the prospective impact of cash-based public health interventions. One possible means of producing an evidence base for policymakers is microsimulation of health and health economic impacts.

In this article, we advance such work by analysing the relationship between income and health among whole adult population using longitudinal data from Understanding Society: The UK Household Longitudinal Study (UKHLS). This study builds on our previous paper [3] that focused on mental health among 16- to 24-year-olds, developing the analytical methods further and extending the analysis to all UK adults.

### Research questions.

We seek to answer two specific, but related, questions:

1. What is the magnitude and shape of association between **average** household income and health among UK adults aged 18 + ?
2. What is the magnitude and shape of association between **changes** in household income and health among UK adults aged 18 + ?

### Objectives.

The objective of the study is to produce data that can be used to microsimulate the prospective health impact of cash transfer schemes that reflect likely UK policies in a UK context. This develops an evidence base that would enable policymakers to assess policies ahead of their introduction and to weigh the cost-benefit more effectively, including whether further research is required.

### A public health crisis associated with financial insecurity

The UK, and much of the wider world, is facing a prolonged period of financial insecurity and increased poverty and inequality due to the cost-of-living crisis. The annual rate of inflation as measured by the Consumer Price Index was above 6% in the UK between February 2022 and September 2023, peaking at 10.7% in November 2022. In response to this, the Bank of England raised the base rate from 0.1% on 15 December 2021 to 5.25% on 3 August 2023 [7]. More than 2.4 million people’s fixed-rate mortgage deals are ending in 2024 and the Resolution Foundation has warned that total annual loan payments are on course to rise by £15.8 bn between mid-2023 and 2026. The average household remortgaging in 2024 faces increased payments of almost £3,000 per year [8]. Meanwhile, strikes in 2023 were at a sustained scale unseen since the 1980s, with many public sector disputes continuing into 2024 [9]. Having experienced severely constrained funding settlements since 2010, various NHS services across the UK are unable to meet accident and emergency, cancer and elective care targets [10], including those that are part of England’s three-year recovery programme [11].

Local authorities, which are responsible for public health and social care, are now declaring effective bankruptcy at an alarming rate [12], with almost one in five council leaders believing their councils are fairly or very likely to be subject to bankruptcy in 2024/25 [13]. Unfortunately, population health and social care needs are only likely to continue to grow, with today’s pressures on the determinants of health contributing to increases in the number and complexity of short, medium and long-term health conditions [14,15] Government healthcare expenditure in the UK in 2022 was already estimated at £230bn, a misleading fall from the elevated figure in 2021 resulting from the COVID-19 pandemic, with a further £52bn financed through non-government healthcare schemes [16]. The King’s Fund estimated total spending on adult social care in 2021/22 of £26.9bn [17], while the Mental Health Foundation and LSE estimated a cost to the UK economy from mental health problems of a minimum of £117.9bn each year, which was largely due to productivity reductions [18]. The Health and Safety Executive found that, in 2022/23, stress, depression or anxiety accounted for 875,000 (49% of all) cases of self-reported work-related ill health and a total of 17.1 million working days lost, which remains higher than in the pre-pandemic period [19].

This trend is reflected in the proportion of the population with a long-standing illness, disability or impairment which causes substantial difficulty with day-to-day activities [15]. This is estimated to have risen from 19% to a high of 24% between 2011/12 and 2021/22, an increase of 3.9m people [20], with an increase from 14.1m in 2019/20 before the pandemic to 16m in 2021/22 [20]. The increase between 2011/12 and 2021/22 has been driven by a rise among working-age adults (16% to 23%) and children (6% to 11%), with the rate for state-pension-age adults remaining the same (45%). This suggests that factors other than an ageing population are at play [20].

There have, undoubtedly, been other social and technological developments that might have contributed to increased rates of long-term health conditions. However, there is clear evidence that socioeconomic factors, including income, are a substantial determinant of health. Starting in 1967, the Whitehall Study of British civil servants first identified a health gradient that affected every grade of employment [21]. This has since been reinforced both by its follow-up study, Whitehall II, and through observational analyses of longitudinal datasets, natural experiments, and trials of cash transfers and other income interventions. For example, associations have been found between income disparities and self-rated health [22–25]; mortality [22,23]; biomarkers [25]; child health and wellbeing outcomes [26]; mental health among children and young people [3,27,28]; and adult mental health [29–31]. There is also evidence [27,32] that both absolute income (the Absolute Income Hypothesis) and income inequality (Relative Income Hypothesis) have a significant impact on health and wellbeing.

Even when considering issues like fuel poverty and financial insecurity, which were historically perceived as relating only to the worst-off, many of those affected in a modern UK context are no longer unemployed or long-term sick. Instead, a larger and larger proportion of the population have become exposed, including many who are in full-time employment or nominal ‘self-employment’ [33], and who receive little or no help from existing conditional benefits. As a result of spikes in energy prices since the Russian invasion of Ukraine and consequent sanctions from Western nations, analysis from the University of York’s Social Policy Research Unit found that ‘some richer households also spend more than 20% of their income on fuel and a quarter of households in fuel poverty are not income poor’ [34].

Forty-four years ago, the Black Report highlighted the role of people’s material conditions on health inequalities and indicated that upstream social spending could address population health in ways that reactive treatment could not [35]. This message was reinforced 30 years later, in 2010, when by the Marmot Review [36], and its update 10 years on, in 2020 [37], which highlighted that trends in inequalities were worsening. UK Governments have in the late 2010s and early 2010s have committed to a prevention agenda [38], which was incorporated into the 2019 NHS England Long Term Plan [39], but much more thought must be put into how to achieve it.

In particular, there have been calls for cash transfer trials, specifically Basic Income, as an upstream intervention that would increase quantity, security and predictability of income to mitigate poverty, inequality and insecurity as social determinants of mental and physical ill-health [2,4]. While welfare systems conditional on means and needs in high-income countries are associated with increased psychological distress prevalence [40] and below average health outcomes [41], Gibson, Hearty and Craig’s [42] systematic review of cash transfer schemes that resemble Basic Income indicated positive impacts on mental and physical health, hospital attendance and health related behaviour, such as alcohol and drug use. We have previously suggested [2] that such outcomes likely emerge due to schemes being ‘insufficient to offset the negative health consequences of severe socioeconomic disadvantage’ [41]; conditionality and assessment inflicting stress [43] and creating perverse incentives for health-diminishing behaviour [2]; and that focusing on the poorest fails to address determinants that affect society as a whole, especially as a greater proportion of the population falls into traps previously experienced by a relatively small minority [36].

This article provides means of estimating some of these impacts.

### Methods

#### Data.

We use data available in 12 waves (2009/11–2020/22) of Understanding Society: The UK Household Longitudinal Study (UKLHS), the entire available dataset at the time of undertaking the analysis [44]. The UKHLS included just under 40,000 households in Wave 1, including around 8,000 from the British Household Panel Survey (BHPS) which ran from 1991 to 2009, and includes socioeconomic, demographic and health data of individuals living in private households in the UK. Data on all members of each household are collected each year, with each wave lasting around 24 months [45]. One person in the household completes a household questionnaire (including questions on household income), and everyone in the household aged 16 + completes an adult questionnaire which covers a range of dimensions, including mental and physical health. It also contains an Ethnic Minority Boost Sample [46] to enable more granular analysis of individual ethnic minority groups.

In order to correct for unequal selection probability, lack of response in the first wave, and attrition at following waves, we used the sample weight variable ‘indinui’ for analysis of individual interview data from multiple waves between waves 1 and 12 for a combined primary sample and Ethnic Minority Boost Sample. As differences in likelihood of selection and response affect the relationship between mental and physical health and other independent variables in the models, we use attrition controls in our regressions to correct for nonresponse [3]. We use a next-wave dummy if the individual participated in the survey the following year and an all-wave dummy if the individual participated in all subsequent waves after starting and a variable that counts the number of waves in which the individual has participated to account for the effect of survivorship bias. These controls are particularly important in the context of a study examining income and health as Contoyannis, Jones and Rice [47]. used BHPS data to show that attrition rates are inversely related to initial health and that attrition is highest among those who start the survey in very poor health, those with lower incomes and those with less formal education (also being particularly high among those who had never married) [3].

We estimate two sets of regressions: one using an unbalanced panel (with the maximum number of individual observations over at least two consecutive waves of the UKHLS), and the other using a balanced panel with only those individuals with complete data for all 12 waves. S1 Table shows the sample size of the unbalanced panel for each wave and gives the size of the balanced panel (which is 6,649 individuals per wave) as a percentage of the sample size for the unbalanced panel in each wave. Overall, the unbalanced panel has just over five times as many observations as the balanced panel. Also, the unbalanced panel has more observations in earlier waves of the UKHLS. Attrition cuts the sample size in later waves and this is only partially compensated for by the boost sample from Wave 7 onwards.

UKHLS data used in this study can be found on the UK Data Service’s website at https://doi.org/10.5255/UKDA-SN-6614-19.

### Imputation

We employed a complete case analysis to address missing values, as our analysis indicated that the data were missing completely at random. This approach allowed us to maintain the integrity of our analyses while minimising bias, as it produces unbiased estimates under missing at random conditions. Furthermore, we applied the analysis to both the balanced and unbalanced panels, and the coefficients on the between-income coefficients are comparable in the balanced and the unbalanced panel model. This implies that the presence or absence of observations with missing waves does not substantially affect the results.

## Measures

Control variables in the analysis include sex, age, ethnicity, disability whether the individual was born in the UK, region, rurality, highest qualification, marital status, employment status and housing tenure. Some of these variables are time-invariant between waves 1 to 12 and some change from wave to wave. These controls seek to address potential confounding factors and enhance the robustness of our findings. Each of the controls can significantly influence both health outcomes and income-earning potential through various mechanisms. Sex may affect health outcomes and income levels through both biological and social mechanisms (or a mix of the two); ethnicity can be associated with varying levels of healthcare access and socioeconomic opportunities; place of birth might impact early-life conditions and cultural factors; disability can directly affect and be affected by health and employment prospects; marital status can influence household resources and social support; age typically correlates with changes in both health and income over the life course. Region of residence in the UK and rural/urban area are correlated with socioeconomic opportunities, deprivation and environmental factors. By including these controls, we aim to isolate the relationship between income and health more effectively, acknowledge broader socioeconomic determinants of health, and identify potential pathways through which income might affect health. Our controls also enhance the generalisability of our results across diverse population subgroups. For highest qualification, employment status and housing tenure, there are potentially substantial overlaps with income, but they have been proposed as having an independent effect on health. This means that we have, if anything, erred on overcontrolling the findings, producing conservative estimates.

### SF-12 (v2).

The SF-12 (v2) survey [48] is a widely used tool to assess an individual’s health-related quality of

life, generating two summary scores: the Physical Component Summary (PCS-12) and the Mental

Component Summary (MCS-12). We include MCS-12 and PCS-12 scores as outcome variables in specifications of the ‘within-between’ model (discussed below). As in our previous study [3], we also create a dichotomous variable for cases of depressive disorder which takes the value of 1 if the individual’s score is ≤ 45·6 and 0 if it is ≥ 45·7 [49]. In the case of the PCS-12, we use a threshold for clinically significant symptoms of a physical health problem of ≤ 50.0 [50]. The items comprising SF-12 can be found in the UKHLS Wave 12 questionnaire [51], with the scoring system found in Ware et al. [48].

### Net equivalised household income.

UKHLS defines a household the same as in UK government surveys, namely, ‘one person living alone or a group of people who either share living accommodation or share one meal a day and who have the address as their only or main residence’, with six months’ continuous residence required ‘implying that students will be included at their term time address, unless living at a hall of residence’ [52].

Net equivalised household income is the sum of net monthly incomes from all household members, adjusted by the OECD-modified equivalence scale [3,53] to account for households of different size and composition. We used net equivalized household income because it enables more accurate comparisons of economic well-being across different household structures. The methodology for calculating net equivalised household income involves the following steps: first, aggregate household income is computed by summing all income sources (this includes earnings, investments, and welfare benefits); second, taxes and mandatory contributions are subtracted to derive net household income; third, the figure is adjusted (equivalised) using the modified OECD equivalence scale, which assigns weights to household members as follows: 1.0 for the first adult, 0.5 for each additional member aged 14 and above, and 0.3 for each child under 14. This weighting system acknowledges the economies of scale in household consumption: larger households can distribute resources more efficiently, thereby requiring proportionally less income per capita to maintain a similar living standard. Net equivalised household income is calculated by dividing net household income by the sum of these weights; finally, this net equivalised household income is attributed uniformly to each household member. The results were uprated using the UK Consumer Prices Index (CPI), to express income in April 2023 prices. Individuals were ranked into income quintiles running from 1 (lowest household equivalent income) to 5 (highest household equivalent income).

### Analyses.

We use two main models for analysis. First, we use a ‘within-between’ model with the continuous SF-12 Mental Component Summary (MCS-12) score or Physical Component Summary (PCS-12) score as regressors. This model combines the effect on health of both an individual’s income in one wave vs their average across waves, and their average across waves compared with the sample average. Second, we use a random-effects logistic regression, with dichotomous health variables created from continuous scores using clinically significant thresholds used to indicate the presence of depressive disorders (MCS-12 ≤ 45.6) or physical health problems (PCS-12 score ≤ 50.0). We also examine different specifications for the within-between model.

The specific model designs are further described below. The code used to analyse the data is available in S2_DataSetup, S3_ContinuousRegressions and S4_LogisticRegressions, which can be found at https://doi.org/10.17605/OSF.IO/SVQFN.

### ‘Within-between’ (Model 1) and random effects logistic regression (Model 2) models.

Building on our previous study [3], we used a ‘within-between’ model with lagged health outcomes to examine the relationship between health (this time both mental and physical) and: i) within-individual variations of income at one time point compared with their average across the waves; and ii) between-individual differences in income averaged across survey waves. As in our previous study, we decided to use this reformulation of the Mundlak model as it is able to retain the flexibility of random effects models while reducing concerns about bias that fixed effects models address [3,54,55]. The ‘within-between’ model offers a balanced solution to the limitations of both random effects and fixed effects models. While random effects provide flexibility in analysing group-level characteristics, they often fall short due to their assumption of covariate exogeneity, which can lead to biased estimators. Conversely, fixed effects models offer unbiased estimators but are less suitable for examining time-invariant group-level characteristics, a limitation particularly relevant in equity research.

The ‘within-between’ specification presents an evidence-based alternative to addressing endogeneity. This method allows for the decomposition of regressors into their time-varying and time-invariant components [55]. Research has shown that the ‘within-between’ model yields identical results (in terms of coefficients and standard errors) to the fixed effects model for the within effect, while also retaining the between effect that fixed effects models cannot measure [55,56]. Furthermore, a key advantage of the ‘within-between’ model is its ability to account for the correlation between group-level effects and explanatory variables. This is achieved by including the group mean into the regression analysis [56].

We also present results from a random effects logistic regression to provide a more intuitive understanding of the impact of different income levels on individuals’ likelihood of having clinically significant mental and physical health symptoms based on established MCS-12 and PCS-12 score thresholds.

### Model 1: continuous health outcome variable (SF-12 scores) – ‘within-between’.

Our models for the determinants of the continuous health outcome variable $h_{it}$ are of the following general form:


$$h_{it}=f\left(y_{it}\right)+\delta^{\prime }h_{it-1}+\eta 'z_{it}+\left(u_{i}+e_{it}\right)$$

This is a random effects GLS regression where:

- $h_{it}$ is the dependent variable, the health outcome (MCS-12 or PCS-12), for individual *i* at time *t*.
- $y_{it}$ is equivalised (before housing costs) household income for individual *i* at time *t*. $f(y_{it})$ is the functional form for the ‘within’ and ‘between’ components of the income variable, specified for four different specifications as set out in the section on ‘income specifications’ below.
- $z_{it}$ is a vector of control variables for individual *i* at time *t*, including sex, ethnicity, whether born in the UK or abroad, limiting long-standing illness or disability, marital status, age (dummy variables in 10-year age groups), highest qualification, economic status, rural/urban indicator, region of residence, and housing tenure.
- Lagged health variables are represented by $h_{it-1}$.
- The residuals $u_{i}$ and $e_{it}$ are assumed to have a mean of zero and be normally distributed.

### Functional form of the income variables $f(y_{it})$ .

We use four different functional forms of the income variable as listed below. In each case the income variable(s) are broken down into a ‘within’ component (average of the income variable over the sample period) and a ‘between’ component (the difference between the income variable at time *t* and the average of the income variable over the sample period).

*Specification 1: log-linear*


$$f\left(y_{it}\right)=\beta \bar{\log y_{i}}+\gamma (\log y_{it}-\bar{\log y_{i})}$$

This is a linear specification using the log of household income as the income measure. $\bar{\log y_{i}}$ is the average of individual *i*’s log income over the sample period, and ($y_{it}-{\bar{\log y}}_{i})$ is *i*’s difference from average log income at time *t*.

This approach enables identification of the effect of differences in income ‘between’ individuals and the ‘within’ effect of an increase/decrease in an individual’s income relative to their average income. The inclusion of a lagged health variable attempts to reduce the impact that reverse causality, health on income, has on the estimates.

*Specification 2: interaction between within and between terms*


$$f\left(y_{it}\right)=\beta \bar{\log y_{i}}+\gamma (\log y_{it}-\bar{\log y_{i})}+\gamma^{B}\log y_{i}(\log y_{it}-\bar{\log y_{i})}$$

This specification allows us to identify if a within-individual increase in (log) income depends on the individual’s average level of income. In other words: does an increase in within-individual income for someone with lower average income have a greater impact than on someone with higher average income?

*Specification 3: log-levels interaction*


$$f\left(y_{it}\right)=\beta \bar{\log y_{i}}+\beta^{A}\bar{\left(y_{i}\log y_{i}\right)}+\gamma^{\prime }\left(\text{log}y_{it}-\bar{\log y_{i}}\right)+\gamma^{A}\left(y_{it}\log y_{it}-\bar{y_{i}\log y_{i}}\right)$$

Specifications 3 and 4 examine whether the estimated relationship between income and health changes at different levels of income. Specification 3 adds the interaction of log income with income in levels. Coefficients $\beta^{A}$ and $\gamma^{A}$capture the log-level interaction terms for the between and within effects respectively. This uncovers differential marginal effects of an increase in income.

*Specification 4: income quintile interactions*


$$f\left(y_{it}\right)=\beta \bar{\log y_{i}}+\beta^{1}q^{1}\bar{\log y_{i}}+\beta^{2}q^{2}\bar{\log y_{i}}+\beta^{3}q^{3}\bar{\log y_{i}}+\beta^{4}q^{4}\bar{\log y_{i}}+$$

$$\gamma (\log y_{it}-\bar{\log y_{i})}+\gamma^{1}q^{1}(\log y_{it}-\bar{\log y_{i})}+\gamma^{2}q^{2}(\log y_{it}-\bar{\log y_{i})}$$

$$+\gamma^{3}q^{3}(\log y_{it}-\bar{\log y_{i})}+\gamma^{4}q^{4}(\log y_{it}-\bar{\log y_{i})}$$

This specification uses interactions between the between and within (log) income terms and dummies for the (within-wave) household income quintiles in the UKHLS data. $q^{1}$, $q^{2}$, $q^{3}$ and $q^{4}$ are the dummy variables for individuals in Quintile 1 (lowest), 2, 3 and 4 respectively, with Quintile 5 the base quintile.

The rationale for specifications 3 and 4 is to test whether the relationship between income and health status varies at different income levels. In specification 3 this is accomplished using a linear interaction with the level of income, and in specification 4 using an interaction with household income quintile dummies.

### Model 2: binary health outcomes (SF-12 scores below threshold level) – random effects logistic regression.

Model 2 estimates the probability of a discrete binary outcome for MCS-12 (scoring ≤ 45.6 – indicative of clinically significant symptoms of depressive disorders – or ≥ 45.7) and PCS-12 (≤50.0 – indicative of clinically significant symptoms of physical health problems – or ≥ 50.1) given the input variable income quintile and the same set of control variables $z_{it}$ as in Model 1 above. To address potential reverse causation bias, we also control for MCS-12 score or PCS-12 score in wave t−1 as shown below. The precise specification is as shown below:


$$\ln\left(\frac{P\left(h_{it}=1\text{|}f(y_{it}\right),z_{it},u_{it})}{P(h_{it}=0|f(y_{it}),u_{it})}=1\right)=\alpha_{i}+B^{\prime }f\left(y_{it}\right)+\delta^{\prime }h_{it-1}+\eta 'z_{it}+u_{it}$$

Where $h_{it}$ is the binary outcome, $\alpha_{i}$ is an individual-specific and time-invariant random effect, *B* is a vector of regression coefficients for the income variables $f(y_{it})$, *δ* is the regression coefficient for health status (PCS-12 or MCS-12 as applicable) in the previous wave t−1, *η* is a vector of regression coefficients for the control variables $z_{it}$ and $u_{it}$ is the error term, uncorrelated across individuals and over time for one same individual. This model was obtained from Wolf [57].

As for Model 1 above, we use four different specifications for the income variables $f(y_{it})$ in Model 2.

The results for Model 2 are presented in S1 Appendix (S5–S8 Tables and S1 Fig and surrounding narrative) as adjusted marginal effects, or differences in probabilities, which are more easily interpretable than log odds.

## Results

### Descriptive statistics

The total analytical sample size for the unbalanced panel over the 12 waves of UKHLS is 80,009 individuals and 429,960 observations, compared with 6,649 individuals and 79,788 for the balanced panel (S1 Table). The UKHLS longitudinal SF-12 analytical sample includes an unbalanced panel (including all individuals aged 18 or over with a full set of covariate information and at least two consecutive waves of data) and a balanced panel (comprising only those individuals with a full twelve waves of data).

As shown in S2 Table, compared to the unbalanced sample, the balanced panel has slightly higher than the unbalanced panel average MCS-12 (50.47 vs 49.50) and PCS-12 (50.18 vs 49.07) scores. Individuals in the balanced panel are also more likely to be between 45 and 74 years of age, white, born in the UK, married (or divorced), have professional or managerial jobs, be employed (or retired), have a degree or other higher education qualification than individuals in the unbalanced sample. Households in the balanced panel have higher than the unbalanced panel average net equivalised household income (£2,411.80 vs £2,169.26) but lower average housing costs (£266.97 vs £328.74). They are also more likely to be owner-occupiers and less likely to be renters.

**Fig 1:**
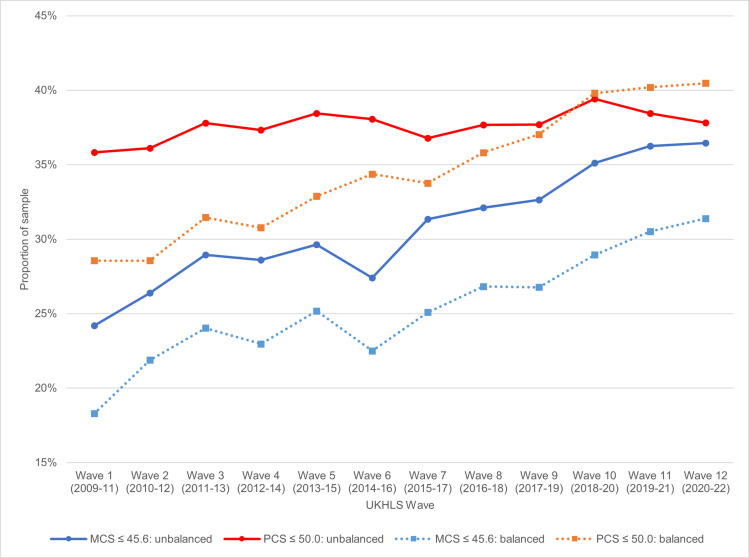
Percentage of respondents with an MCS-12 score of≤ 45.6 indicating clinical depressive disorder and with a PCS-12 score of ≤ 45.6 indicating a physical health problem by wave of interview: unbalanced and balanced panels.

S3 and S4 Tables show the number and percentage of respondents with an MCS-12 of ≤ 45.6 and PCS-12 of ≥ 50 by each wave, for the unbalanced and balanced panels respectively. Overall, 25.37% of respondents in the balanced panel are at or below the threshold of 45.6 for MCS-12 compared to 30.76% of respondents in the unbalanced panel. For the PCS-12, 34.47% of respondents in the unbalanced panel are at or below the threshold of 50 compared to 37.62% of respondents in the balanced panel. The proportion of participants meeting the clinical threshold scores rises significantly over time in both samples. We believe that this reflects a genuine trend of increasing rates of depressive disorders and physical health problems, as highlighted in the Introduction, though there may be other sampling issues at play too. Fig 1 summarises MCS-12 and PCS-12 scores across 12 waves.

#### Violin plots.

**Fig 2:**
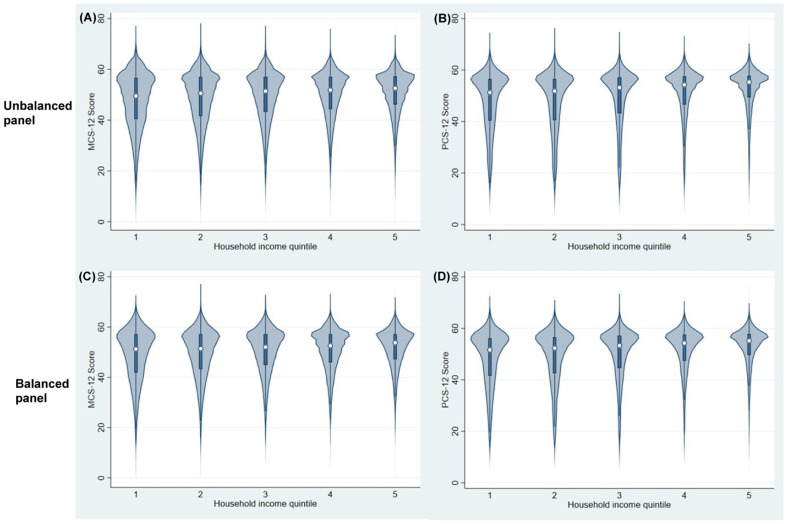
Violin plots of MCS-12 and PCS-12 scores by net equivalised household income quintiles: unbalanced and balanced panels.

Fig 2 shows violin plots of MCS-12 and PCS-12 scores across the five quintiles in net equivalised household income distribution for the unbalanced and balanced panels. Panels (A) and (C) include all the observations across all waves. The median MCS-12 score for all groups in panel (A) varies between 49.5 for the lowest quintile and 52.5 for the top quintile. The median for all groups in panel (C) varies between 50.8 and 55.4 and increases in each subsequent income quintile. Panels (B) and (D) show the violin plots for the balanced panel sample. In both case the median score increases monotonically for richer quintiles, going from 51.2 in quintile 1 to 53.8 in quintile 5 for MCS-12 scores in (B), and from 51.6 in quintile 1 to 55.1 in quintile 5 for PCS-12 scores in (D).

### Regression results

### Model 1: Continuous regressions for MCS-12 and PCS-12 scores

#### Specification 1 (log-linear): unbalanced panel.

Table 1 presents the full set of regression results from the ‘within-between’ model for MCS-12 and PCS-12 (Model 1) for the unbalanced panel sample, using a log-linear specification for household income (Specification 1), with the log of the individual’s net equivalised household income quintile as input variable. The left-hand column shows the results for the MCS-12 score and the right-hand column shows the results for the PCS-12 score.

**Table 1:** Within-between Model 1 (MCS-12 and PCS-12 scores): Specification 1 (log-linear incomes), full regression results: unbalanced panel.

	MCS-12 co-efficient (SE)	PCS-12 co-efficient (SE)
**Between-Income (log)**	0.761*** (0.048)	0.611*** (0.041)
**Within-Income (log)**	0.059 * (0.035)	-0.139*** (0.031)
**Constant term**	22.031*** (0.381)	21.145*** (0.320)
**Controls:**		
**SF-12 Score (t-1)**	0.387*** (0.002)	0.490*** (0.002)
**Sex (Female)**	-1.082*** (0.039)	-0.183*** (0.033)
**Ethnicity:**		
Mixed	-0.583*** (0.082)	-0.213 * (0.125)
Asian	-0.025*** (0.082)	-1.286 (0.069)
Black	1.097*** (0.116)	-0.159 * (0.097)
Other	-0.098 (0.246)	-0.827*** (0.206)
**Born in UK**	-0.272*** (0.064)	0.266*** (0.053)
**Limiting long-standing illness or disability**	-2.023*** (0.036)	-4.441*** (0.032)
**Marital status:**		
Married or in civil partnership	0.705*** (0.052)	-0.185*** (0.044)
Divorced or separated	0.011 (0.071)	-0.392*** (0.059)
Widowed	0.605*** (0.094)	-0.790*** (0.078)
**Age:**		
25–34	-1.111*** (0.075)	-1.292*** (0.063)
35–44	-0.900*** (0.079)	-1.883*** (0.067)
45–54	-0.450*** (0.081)	-2.608*** (0.067)
55–64	0.461*** (0.086)	-3.329*** (0.073)
65–74	1.117*** (0.104)	-3.727*** (0.089)
75+	1.256*** (0.119)	-5.507*** (0.102)
**Highest qualification:**		
Degree	-0.206*** (0.075)	1.806*** (0.063)
Other higher education	0.111 (0.084)	1.282*** (0.068)
A-level or equivalent	0.139 * (0.074)	1.272*** (0.062)
GCSE or equivalent	0.276*** (0.073)	1.021*** (0.061)
Other qualifications	0.259*** (0.084)	0.563*** (0.070)
**Economic status:**		
Employee	2.713*** (0.066)	2.490*** (0.057)
Self-employed	3.155*** (0.088)	2.669*** (0.075)
Looking after family	2.278*** (0.093)	1.998*** (0.080)
Unemployed	0.515*** (0.093)	1.937*** (0.080)
Retired	3.327*** (0.089)	1.525*** (0.076)
**Lives in rural area**	0.402*** (0.045)	0.206*** (0.037)
**English Region:**		
North West	0.224** (0.113)	-0.096 (0.094)
Yorkshire and the Humber	0.246** (0.117)	0.047 (0.097)
East Midlands	0.331*** (0.119)	0.123 (0.099)
West Midlands	0.007 (0.117)	0.007 (0.098)
East of England	0.360*** (0.116)	0.207** (0.096)
London	0.249** (0.117)	0.279*** (0.098)
South East	0.286** (0.111)	0.333*** (0.092)
South West	0.296** (0.116)	0.258*** (0.097)
**Country:**		
Scotland	0.474*** (0.116)	0.117 (0.096)
Wales	-0.041 (0.120)	-0.242*** (0.100)
Northern Ireland	0.638*** (0.123)	-0.474*** (0.102)
**Housing tenure:**		
Owner-occupier	0.490*** (0.057)	0.580*** (0.048)
Social renter	-0.480*** (0.069)	-0.714*** (0.058)
**Observations**	315,093	315,093
**Individuals**	58,729	58,729
**R-squared:**		
Within	0.0027	0.0284
Between	0.6233	0.8113
Overall	0.3619	0.6111

*p <  0.10,

**p <  0.05,

***p <  0.01

The results show that the difference between individuals’ average levels of income is a positive and highly significant predictor of mental and physical health. In column 1, the coefficient for the between-effect is 0.76, indicating that individuals with higher incomes have higher MCS-12 scores, controlling for other factors. In column 2, the coefficient for the between-effect is 0.61, indicating that individuals with higher income quintiles had higher PCS-12 scores on average.

For the within-effects (the variations for each person in each wave compared to their average income across all waves), the estimated relationships look quite different for the MCS-12 and PCS-12. For the MCS-12, the within-effect coefficient is not significant at the 5% level and only 0.06, suggesting that within-person changes in income are associated with only small, marginally significant increases in MCS-12 scores. These results suggest that income quintile is more strongly associated with MCS-12 score when comparing individuals between different income quintiles, rather than looking at within-person changes. For the PCS-12 the within-effect coefficient is significant but negative at -0.14, suggesting that within-person changes in income quintile were associated with a small *decrease* in PCS-12 scores.

Turning to the control variables, SF-12 score in the previous wave has a strong positive association with SF-12 score in the current wave (which is unsurprising). Being female is associated with a lower MCS-12 and PCS-12 score than being male controlling for other factors, with the relationship being considerably larger for the MCS-12 than the PCS-12. Mixed and Asian ethnicities are associated with lower MCS-12 compared to white (the control group), whereas black ethnicity is associated with higher PCS-12 compared to white. People who are born in the UK have lower MCS-12 scores but higher PCS-12 scores on average than those born outside the UK. People with a limiting long-standing illness or disability have lower MCS-12 and PCS-12 scores than those without, controlling for other factors. The effect is larger for PCS-12 scores (just over 4 points lower on average for those with a limiting long-standing illness or disability compared to those without) but is still large for MCS-12 scores (just over 2 points lower on average for those with compared to those without).

Looking at the relationship between PCS-12 and MCS-12 scores and marital status, married people have higher MCS-12 but lower PCS-12 on average than the base group (single never married), controlling for other factors. Divorced people have lower PCS-12 than the base group. Widowed people have lower PCS-12 but higher MCS-12 than the base group.

The age effects show that compared to the base category (18- to 24-year-olds), MCS-12 score is lower on average for 25- to 54-year-olds but higher for individuals aged 55 + . PCS-12 score for older groups is lower on average than for 18- to 24-year-olds, with the average drop in PCS-12 scores increasing with age.

Looking at highest qualifications, people with degrees have slightly worse MCS-12 scores on average compared to the base category (those with no qualifications). All other qualification groups have higher MCS-12 scores on average than those with no qualifications, controlling for other factors. All qualification groups have higher PCS-12 scores than the base group of those with no qualifications.

The estimated effects by economic status show that employees, retired people, students, unemployed jobseekers and people looking after family all have significantly higher MCS-12 and PCS-12 scores than the base group (working age inactive people). Employees and self-employed people have the highest average PCS-12 scores controlling for other factors, while retired people and the self-employed have the highest average MCS-12 scores controlling for other factors.

Examination of the pattern of coefficients on the regional dummies shows that compared to the base category (North East England) all other English regions, Scotland and Northern Ireland have significantly higher MCS-12 scores. London and the other southern regions (East of England, South East and South West) have higher average PCS-12 scores than North East England, while Wales and Northern Ireland have lower average PCS-12 scores.

Finally, looking at the coefficients on housing tenure, people living in owner-occupied homes have significantly higher MCS-12 and PCS-12 scores than private renters (the base category), while social renters have significantly lower MCS-12 and PCS-12 scores than private renters on average.

#### Specification 1 (log-linear): balanced panel.

Table 2 presents full results from Specification 1 of the within-between model using the balanced panel. The coefficients on the between-income effects look similar to the unbalanced panel model in Table 1, with 0.66 for MCS-12 and 0.59 for PCS-12. The within-income effects are insignificant in the MCS-12 model and only significant at the 10% level in the PCS-12 model. Most of the estimated coefficients for gender and ethnicity for the balanced panel look similar to those for the unbalanced panel, although the negative coefficient for Asian ethnicity is larger in the MCS-12 model for the balanced panel. The coefficients for the ‘born in the UK’ dummy, limiting long-standing illness or disability, marital status and age look similar to those for the unbalanced panel, although the larger standard errors in the smaller balanced panel sample means that some of the coefficients on age group in the MCS-12 model are not statistically significant.

**Table 2:** Within-between Model 1 (MCS-12 and PCS-12 scores): Specification 1 (log-linear incomes), full regression results: balanced panel.

	MCS-12 co-efficient (SE)	PCS-12 co-efficient (SE)
**Between-Income (log)**	0.662^***^ (0.078)	0.591^***^ (0.067)
**Within-Income (log)**	-0.021 (0.072)	-0.121 * (0.063)
**Constant term**	14.497^***^ (0.644)	13.119^***^ (0.562)
Controls:		
**SF-12 Score (t-1)**	0.558^***^ (0.003)	0.624^***^ (0.003)
**Sex (Female)**	-0.726^***^ (0.057)	-0.188^***^ (0.049)
**Ethnicity:**		
Mixed	-0.305 (0.254)	-0.342 (0.221)
Asian	-0.584^***^ (0.167)	-0.713^***^ (0.146)
Black	0.955^***^ (0.232)	0.436^**^ (0.202)
Other	-1.006 ^*^ (0.517)	-0.482 (0.451)
**Born in UK**	-0.371^***^ (0.115)	0.240^**^ (0.101)
**Limiting long-standing illness or disability**	-1.253^***^ (0.060)	-3.550^***^ (0.056)
**Marital status:**		
Married or in civil partnership	0.493^***^ (0.082)	-0.129 ^*^ (0.072)
Divorced or separated	-0.004 (0.108)	-0.207^**^ (0.094)
Widowed	0.438^***^ (0.147)	-0.333^***^ (0.128)
**Age:**		
25–34	-1.023^***^ (0.236)	-1.016^***^ (0.206)
35–44	-0.804^***^ (0.232)	-1.445^***^ (0.203)
45–54	-0.357 (0.231)	-1.885^***^ (0.202)
55–64	0.396 ^*^ (0.234)	-2.349^***^ (0.205)
65–74	0.891^***^ (0.252)	-2.641^***^ (0.220)
75 +	1.070^***^ (0.272)	-3.589^***^ (0.238)
**Highest qualification:**		
Degree	-0.459^***^ (0.132)	1.231^***^ (0.115)
Other higher education	-0.265 ^*^ (0.137)	0.808^***^ (0.119)
A-level or equivalent	-0.161 (0.134)	0.894^***^ (0.117)
GCSE or equivalent	-0.035 (0.132)	0.534^***^ (0.115)
Other qualifications	-0.060 (0.147)	0.352^***^ (0.129)
**Economic status:**		
Employee	2.990^***^ (0.160)	3.628^***^ (0.142)
Self-employed	3.263^***^ (0.184)	3.687^***^ (0.162)
Looking after family	2.725^***^ (0.205)	3.239^***^ (0.180)
Unemployed	1.464^***^ (0.242)	3.386^***^ (0.213)
Retired	3.633^***^ (0.180)	3.141^***^ (0.158)
**Lives in rural area**	0.066 (0.065)	0.141^**^ (0.056)
**English Region:**		
North West	0.064 (0.148)	-0.125 (0.129)
Yorkshire and the Humber	0.233 (0.157)	0.078 (0.137)
East Midlands	0.315^**^ (0.155)	0.258 ^*^ (0.135)
West Midlands	-0.098 (0.153)	-0.043 (0.134)
East of England	-0.008 (0.149)	0.163 (0.131)
London	-0.075 (0.164)	0.045 (0.143)
South East	0.074 (0.142)	0.215 ^*^ (0.124)
South West	0.071 (0.148)	0.214 ^*^ (0.129)
**Country:**		
Scotland	0.142 (0.159)	0.027 (0.138)
Wales	-0.082 (0.188)	0.126 (0.164)
Northern Ireland	0.081 (0.210)	-0.526^***^ (0.183)
**Housing tenure:**		
Owner-occupier	0.527^***^ (0.108)	0.361^***^ (0.094)
Social renter	-0.521^***^ (0.138)	-0.510^***^ (0.121)
**Observations**	73,139	73,139
**Individuals**	6,649	6,649
**R-squared:**		
Within	0.0127	0.0573
Between	0.9198	0.9528
Overall	0.3959	0.6004

* p <  0.10, ** p <  0.05, *** p <  0.01

The coefficients on highest qualification in the balanced panel MCS-12 model look quite different from the unbalanced panel for GCSE and other qualifications, both of which are positive and significant in the unbalanced panel but insignificant (and negative) in the balanced panel. The estimates for highest qualification in the PCS-12 balanced panel model look more similar to the unbalanced panel model. The employment status and housing tenure estimates for the balanced panel models look reasonably similar to the unbalanced panel model. Finally, none of the estimated coefficients for region or country dummies are statistically significant at the 5% level for the balanced panel models (except for the East Midlands in the MCS-12 model).

For the other three model specifications we do not present the full set of regression results because they look very similar to specification 1. Instead, we present the within- and between-income coefficients only.

#### Specification 2: within-between interactions.

Table 3 below shows the result from Specification 2 of model 1 (which includes an interaction term for within-between incomes). The interaction terms are not statistically significant for either the unbalanced or balanced panel samples, for either the MCS-12 or PCS-12 score models. Furthermore, the inclusion of the within-between interaction term means that the within-income terms – which had been significant at the 1% level in the PCS-12 score unbalanced panel model (spec 1) and at the 10% level in the PCS-12 score balanced panel model spec 1, are no longer statistically significant. This suggests that the negative association between within-income and PCS-12 score in the unbalanced panel in Specification 1 (in Table 1 above) is not robust to the inclusion of the within-between interaction term.

**Table 3:** Results from Model 1, Specification 2 (within-between interactions).

	Unbalanced panel coefficients (SE):		Balanced panel coefficients (SE):	
**Log income coefficients:**	**MCS-12**	**PCS-12**	**MCS-12**	**PCS-12**
Between-income	0.761*** (0.048)	0.611*** (0.041)	0.661*** (0.077)	0.592*** (0.067)
Within-income	-0.444 (0.333)	0.014 (0.288)	-1.524 (0.993)	0.750 (0.866)
Within-between interaction	0.069 (0.045)	-0.039 (0.040)	0.202 (0.133)	-0.117 (0.116)
**Constant term**	22.032*** (0.381)	21.146*** (0.320)	14.500*** (0.643)	13.117 (0.562)
**Observations**	315,093	315,093	73,139	73,139
**Individuals**	58,729	58,729	6,649	6,649
**R-squared:**				
Within	0.0027	0.0284	0.0127	0.0573
Between	0.6226	0.8114	0.9198	0.9528
Overall	0.3619	0.6111	0.3959	0.6004

*p <  0.10, ^**^ p <  0.05,

***p <  0.01

#### Specification 3: Log-level interactions.

Table 4 shows the results for the income variables from specification 3 of Model 1 where the income terms include an interaction between the level of incomes and log incomes (to establish whether the income effects vary according to the level of income). The coefficients on the log-level interaction terms in Table 4 are multiplied by 100,000 to make them easier to read at 3 decimal places. The results for the MCS-12 models show that the between-incomes log-level interaction is significant at the 10% level (and positive) for the unbalanced model, and insignificant for the balanced panel model. The within-income terms are insignificant in both MCS-12 score models. For the PCS-12 score models the log-level interaction term is significant at the 1% level (and negative) for the unbalanced sample but insignificant for the balanced panel. The within-income log-level interactions are insignificant in both PCS-12 models.

**Table 4:** Results from Model 1, Specification 3 (log-level interactions).

	Unbalanced panel coefficients (SE):		Balanced panel coefficients (SE):	
**Income coefficients:**	**MCS-12**	**PCS-12**	**MCS-12**	**PCS-12**
**Between-income:**				
Log	0.685*** (0.063)	0.458*** (0.053)	0.558*** (0.135)	0.492*** (0.118)
Log x levels (x1000)	0.345* (0.189)	-0.714*** (0.158)	0.440 (0.471)	0.421 (0.411)
**Within-Income:**				
Log	0.056 (0.036)	-0.139*** (0.031)	-0.021 (0.072)	-0.122* (0.063)
Log x levels (x1000)	0.105 (0.153)	-0.105 (0.132)	-0.113 (0.386)	-0.009 (0.036)
**Constant term**	22.534*** (0.467)	22.172*** (0.392)	15.202*** (0.992)	13.117*** (0.562)
**Observations**	315,093	315,093	73,139	73,139
**Individuals**	58,729	58,729	6,649	6,649
**R-squared:**				
Within	0.0027	0.0284	0.0127	0.0573
Between	0.6226	0.8114	0.9198	0.9528
Overall	0.3619	0.6111	0.3959	0.6004

*p <  0.10, ^**^ p <  0.05,

***p <  0.01

#### Specification 4: income quintile interactions.

**Fig 3:**
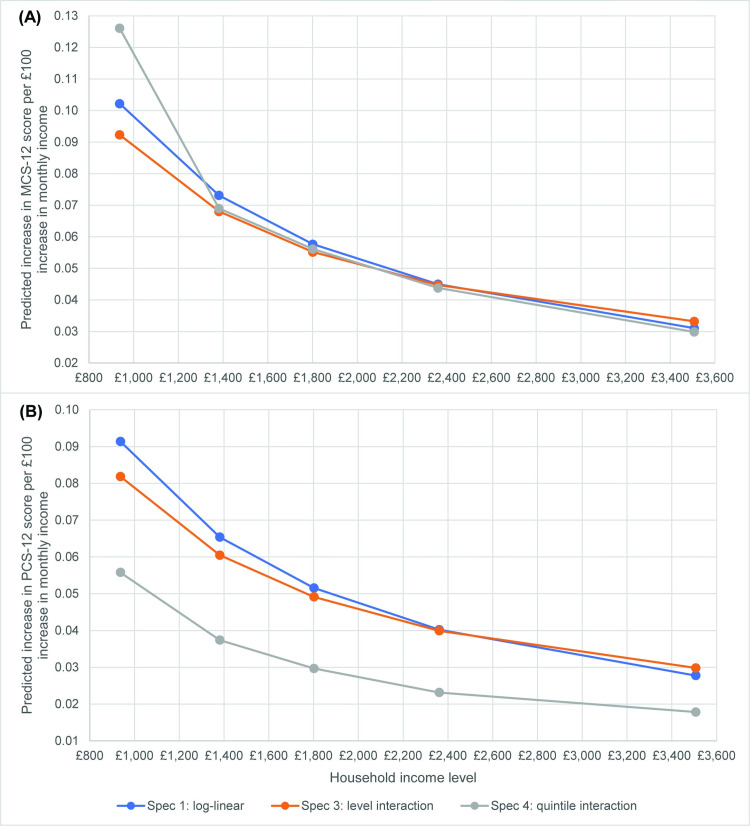
Predicted effect of £100 per month increase in equivalised household net income on MCS-12 and PCS-12 scores, for Model 1, Specifications 1, 3 and 4: balanced panel Note: Specification 2 is omitted because the coefficients on the between-income variables are almost identical to Specification 1 (to within.001).

Finally in this section, Table 5 shows the results from specification 4 where the between-income and within-income effects are interacted with income quintile dummy variables. For the MCS-12, the results for the unbalanced panel show that the between-income quintile dummies are negative and significant compared to the base quintile (quintile 5, i.e., the top income quintile). This suggests a slightly weaker relationship between log income and MCS-12 for lower incomes compared to the top quintile. Note however that – as shown graphically in Fig 3 below – an increase in the *level* of income (say, an extra £100 per month) has a larger positive impact on MCS-12 score at lower incomes than at higher incomes. In the balanced panel the quintile dummy effects for MCS-12 are smaller and statistically insignificant.

**Table 5:** Results from Model 1, Specification 4 (quintile interactions).

	Unbalanced panel coefficients (SE):		Balanced panel coefficients (SE):	
**Income coefficients:**	**MCS-12**	**PCS-12**	**MCS-12**	**PCS-12**
**Between-income:**				
Log	0.311*** (0.013)	0.422*** (0.062)	0.636*** (0.136)	0.380*** (0.119)
Log x quintile 1	-0.087*** (0.013)	-0.025** (0.011)	0.018 (0.026)	-0.019 (0.022)
Log x quintile 2	-0.082*** (0.011)	-0.048*** (0.008)	-0.012 (0.021)	-0.042** (0.018)
Log x quintile 3	-0.048*** (0.009)	-0.049*** (0.008)	0.008 (0.016)	-0.037*** (0.014)
Log x quintile 4	-0.026*** (0.007)	-0.025*** (0.006)	0.011 (0.012)	-0.014 (0.011)
**Within-Income:**				
Log	-0.040 (0.093)	-0.244*** (0.080)	0.241 (0172)	-0.192 (0.151)
Log x quintile 1	0.043 (0.109)	0.207** (0.093)	-0.241 (0.205)	0.115 (0.178)
Log x quintile 2	-0.579*** (0.153)	-0.093 (0.147)	-0.432 (0.340)	-0.188 (0.297)
Log x quintile 3	-0.922*** (0.166)	-0.261* (0.142)	-0.426 (0.320)	-0.301 (0.278)
Log x quintile 4	-0.748*** (0.160)	-0.558*** (0.137)	-0.605** (0.299)	-0.391 (0.261)
**Constant term**	25.815*** (0.597)	22.813*** (0.507)	14.644*** (1.147)	14.908** (1.001)
**Observations**	315,093	315,093	73,139	73,139
**Individuals**	58,729	58,729	6,649	6,649
**R-squared:**				
Within	0.0027	0.0285	0.0127	0.0573
Between	0.6226	0.8116	0.9198	0.9529
Overall	0.3620	0.6112	0.3959	0.6005

*p <  0.10,

**p <  0.05,

***p <  0.01

In the PCS-12 model, there are negative coefficients on the income quintile dummies compared to quintile 5 in the unbalanced panel model. In the balanced panel, only quintiles 2 and 3 are significant (and negative) compared to quintile 5. Meanwhile, the within-income quintile dummies are significant and negative in quintiles 2, 3 and 4 for the unbalanced MCS-12 model but mostly insignificant for the other models.

#### Visualising coefficients on between-incomes for Model 1, Specifications 1, 2 and 4.

The combination of income coefficients for Specification 2, 3 and 4, and the fact that log income is used for the main income measures, makes it harder to interpret the size of the predicted income coefficient in the regressions for Model 1. To make it easier to interpret the predicted effects, Fig 3 shows the predicted effects of a £100 per month increase in equivalised household net income on MCS-12 (panel A) and PCS-12 (panel B) scores in the balanced panel. Note that these panels show the impact of an increase in the between-income effects only because the within-income effects are not statistically significant in most of the balanced panel specifications. Effectively the graphs show the impact of a sustained increase in income of £100 per month across all 12 waves of the UKHLS sample (or an equivalent increase in average income across waves, differently distributed over time). Panel (A) shows that for the lowest income quintile, an increase in income of £100 per month is associated with an increase in MCS-12 score of around 0.10 (for Spec 1), 0.09 (for Spec 3) and just over 0.12 (for Spec 4). For higher income quintiles there is also a positive impact of increased income, but the magnitude falls as income rises. For income quintile 4 the MCS-12 increase is around 0.045 and for the top income quintile it is just over 0.03. The implication is that redistributing income from richer to poorer households raises the overall mental health of the population. Panel (B) shows that for the lowest income quintile, an increase in income of £100 per month is associated with an increase in PCS-12 score of around 0.09 for Spec 1 and 0.08 for Spec 3. As with the MCS-12 scores in Panel (A), for higher income quintiles there is also a positive impact of increased income, but the magnitude falls as income rises. For income quintile 4 in Specifications 1 and 3 the MCS-12 increase is around 0.04 and for the top income quintile it is around 0.03. For Specification 4, The implication is that redistributing income from richer to poorer households raises the overall mental health of the population.

#### Random effects logistic panel regressions (Model 2): Probability of MCS-12 and PCS-12 score below critical thresholds.

S5–S8 Tables, S1 Fig and surrounding narrative in S1 Appendix show the results from the random effects logistic panel regressions and the probability of having a score below the MCS-12 and PCS-12 clinically significant thresholds. In summary, they confirm the findings from the continuous regressions, in showing that average income quintile is inversely and monotonically associated with the probability of having clinically significant symptoms of depressive disorders and physical health problems.

## Discussion

Our analysis of 12 waves of UKHLS data shows that increases in an individual’s income compared with their average income and a higher income compared with the sample average is associated with better mental health and better functional physical health. However, as with the declining rate of marginal utility, the impact of an increase in household income on mental and physical health is lower at higher incomes for a standard increase in household income (e.g., £100 per month). This suggests that redistribution from high-income households to lower income households would increase the average physical and mental health of the population, other things being equal. Using the logistic regression, we find that average income quintile is inversely and monotonically associated with the probability of having clinically significant symptoms of depressive disorders and physical health problems. As with the results from the within-between model, the associations between a given increase in income and the change in probability of experiencing mental or physical health problems is smaller in absolute terms at higher points in the income distribution. We suggest that the results support the policy implications derived from the Relative Income Hypothesis, as average population health should improve through redistribution from the top end to the bottom and other points in between.

### Comparison with previous studies

The findings broadly reflect those from previous work by this project team, which included analysis of 10 waves (2009/11-2018/20) of UKHLS data with regard to MCS-12 mental health among 16- to 24-year-olds in identifying a significant gradient based on household income [3]. This was also supported by our analysis [28] of Millennium Cohort Study and Next Steps data based on household income as well as composite indices of deprivation and a subjective measure of financial strain and SMFQ at age 14, Kessler-6 at age 17 and GHQ-12 at age 25 measures of mental health.

The findings are similar to those of Thomson et al.’s [31] systematic review which narratively synthesised 136 papers and meta-analysed 86 in relation to the impact of income changes on mental health and wellbeing among working-age adults in countries with all levels of income. They found that an income increase that lifted individuals out of poverty was associated with larger improvements than other increases. In another study looking specifically at nine waves (2009–19) of UKHLS and the effects of poverty on the mental health of adults aged 25-64, Thomson et al. [58] found that evidence to suggest that ‘moves into poverty account for just over 6% of the burden of CMD in the UK working-age population, with larger effects in women.’

Akanni, Lenhart and Morton’s used a fixed-effects model for health and wellbeing outcomes using 11 waves (2009/11–2017/19) of UKHLS data which found that ‘increased household income is associated with an increased likelihood of reporting excellent general health outcome, the least distress in mental health outcomes, and more satisfaction with life’ [29] Using cross-sectional regressions, they also found that ‘stability in household disposable income is positive and significantly associated with the considered health and wellbeing outcomes’, and that ‘length of time individuals endure low (high) income reduces (increases) their odds ratio of reporting improved self-rated health and subjective wellbeing’ [29]

On the other hand, Sinha et al. [59] analysed UKHLS and BHPS data and found that the income, as part of the socioeconomic gradient in most of the health measures used accounted for only 3.8% of total deprivation. They also found evidence of a ‘systematic deprivation gradient for BMI, waist circumference, heart rate, C-reactive protein and HbA1c’ [59], which are associated with the development of a number of health conditions. These findings with regard to multidimensional deprivation reflect previous findings from some of the authors of this paper, which suggested a much steeper gradient between an index of deprivation and mental health among people aged 14–25 than objective income alone [28].

Analysing eight waves of UKHLS (2009-2018) data, Anderson [60] used cross-sectional and longitudinal structural equation models to examine inequality between income and GHQ-12 mental health. Using cross-sectional models, he found that inequality become significant at age 25 among both men and women, increasing until early 30s and persisting, with volatility, until the mid-50s. It then declines from the late 50s. In this model, he found that lower inequality when controlling for employment. However, using longitudinal models, he found that while leaving employment is negatively associated with subsequent mental health ‘neither this mechanism nor the reverse health selection out of employment – can account for growth in mental health inequality. Rather, only unobserved heterogeneity between individuals accounts for a substantial portion of this growth – around a third’ [60].

Benzeval et al. summarised conflicting accounts with regard to income and health by finding a ‘strong theoretical consensus that money does matter for health and the relationship is a positive one’, but also that there is ‘less clarity regarding the particular role of income as a health determinant or the mechanisms by which income modification interventions might affect health’ [61].

A limitation of our analysis is that it uses UK data and therefore speaks to dynamics at play in a high-income country with an established system of universal healthcare and cash benefits. However, the Global Financial Crisis, austerity policies, Brexit, the COVID-19 pandemic, the war in Ukraine and the cost-of-living crisis have all had a substantial negative effect on the economy, real-terms incomes, wealth inequality, income security and destitution [62]. While the UK’s commitment to austerity and concomitant increases in inequality from 2010 is an extreme version of an approach shared by many high-income countries, evidence [63] suggests that income inequality negatively effects health in nations at pretty much all stages of development. The suggestion that the very worst-off in society must receive the greatest focus in increasing incomes to exceed a threshold below which outcomes are disastrously worse, is, of course, very reasonable in both high-income and low-middle income countries and supported by our findings. However, there are also very substantial gains to be made among those at any point below very top of the distribution and at little cost to those at the top.

### Subjective measures of financial circumstances

We have previously proposed that financial strain may be an important subjective measure of socioeconomic status that does not always track net equivalised household income exactly.^3^ In relation to this, French analysed data from the BHPS waves 11–18 and UKHLS waves 2–5 using a model that proposes financial strain as being driven by the following:

1. Permanent income is insufficient to meet basic needs, putting the household under financial strain;
2. Permanent income shocks cause consumption levels to be revised and such revisions cause strain; or
3. Households are liquidity constrained and cannot smooth income over time [64].

He found ‘that financial strain worsens individual mental health and general health status even when controlling for individual heterogeneity and potential reverse causation’, that ‘shocks to how we view our financial situation are more important for subjective financial well-being than the level of income or being liquidity constrained’ and that this might explain why the ‘reassessment process [from Incapacity Benefit/Severe Disablement Allowance to Employment and Support Allowance] increased the probability of feeling financially strained by 7.9 percentage points’ [64].

Given the number of results we present here, we do not include analysis using a subjective measure of financial circumstances, such as financial strain which we used in our previous study on 16- to 24-year-olds and mental health [3]. However, we believe that such measures are an important, and straightforward, means of measuring respondents’ assessment of their day-to-day financial position and that there is good evidence of its association with health. Further analysis of the association between subjective measures of financial circumstances (whether how well participants feel they are managing financially, or, as we have used in more recent work [62], participant-assessed risk of destitution) would provide a more comprehensive understanding of the potential mediators between money and health.

The use of Net Equivalised Household Income as a measure averaged across the country may be a limitation in our analysis. Average salaries vary significantly in different regions of the UK, and the importance of subjective social status for health outcomes [65] means that our analysis may underestimate differences between income levels at a local level that occur below the very top end of the national distribution.

### Reverse causality

Our analysis is observational and therefore cannot fully address assertions from some that the direction of causation within the associations we identify is more likely to be, or more prominently, from health to income. However, French’s [64] analysis, along with Nettle and colleagues’ Changing Cost of Living Study (see below) [66], provide support for inferring direction of causation from income, and in particular level of financial strain, to health.

While it is relatively clear that income has the potential to impact health, through inability to satisfy basic needs for example, health also has the potential to impact income and employment. Bias in quantifying the relationship, particularly in a causal sense, between income and health might occur because:

-  There may be unobservable variables, here referring to factors that simultaneously affect income and health, such as early-life exposures, or environmental factors, that we cannot see or measure. For example, malnutrition or exposure to pollution during childhood can lead to developmental delays, health conditions that also affect neuro development and income generation capacity later in life. Our within-between model introduces fixed effects which might partially account for unobservable bias. By repeatedly measuring the same individuals over time, we address time-invariant unobserved variables that may influence both the independent variable (e.g., income) and the dependent variable (e.g., health) simultaneously.
-  There is a bidirectional relationship between income and health, as was the conclusion with regard to income and mental health in a report for the Health Foundation, by Wilson and Finch [67]. Changes in income can influence health, but at the same time, changes in health can also impact an individual’s income-generating capacity. Individuals with poor health may have to reduce their economic activity, for example by working fewer hours, entering retirement early or taking sick leave, resulting in lower income levels.

**Fig 4:**
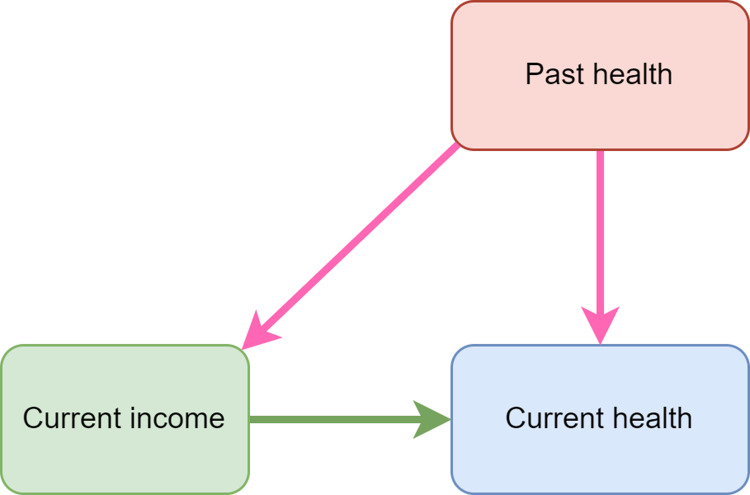
DAG diagram of the relationship between current income, past health and health.

Regarding the last point, we seek to address the potential for overestimating the causal effect of household income on health by controlling for last year’s physical and mental health. As in our previous paper, our DAG diagram (Fig 4) illustrates this through the pink causal pathways. However, this assumes a lag of about a year between physical and mental health changes and effects on earnings. If they occur within the year, for example if an individual experiences a large and significant physical health condition that leads to their leaving a physically demanding job very rapidly, our model does not address potential resulting bias.

However, it seems plausible that physical health, in particular, is determined over a much longer period. Indeed, Blakely et al. [68] analysed data for 213,695 people aged 15 years and older sampled by the 1995 and 1997 Current Population Survey (CPS) in the United States and found that ‘income inequality up to 15 years previously may be more strongly associated with self rated health than income inequality measured contemporaneously, for people aged 45 years and older at least.’ Zheng’s [69] analysis of US National Health Interview Survey data covering 1986-2004 alongside follow-up mortality data from 1986-2006 found that there was a lag in the association between income inequality and individual mortality risk of five years, peaking at seven and diminishing after 12.

We explored the use of alternative panel models, and when we added a three-year lag to the within component of the within-between model (shown in S9 and S10 Tables), we found little difference in estimates compared with specification 1 of Model 1 (which includes the contemporaneous within-income component, shown in Table 2 in the main paper). Conceptually, there may be good reason for this. With regard to the within-between model, the within-individual component may be similar to a temporary income shock as it measures the effect of a difference between an individual’s income in one wave compared to their average. The between-individual component, on the other hand, is likely to measure effects that are much more enduring, less skewed by macroeconomic changes in income across all participants and perhaps built over a much longer period. This measures effects that are more akin to permanent income changes. In addition, they evoke studies demonstrating the health effects of subjective social status [70] beyond those resulting from more objective measures, including for those above the poverty line.

Some of the authors of this report have also been part of a quasi-experimental study entitled the Changing Cost of Living [66], which tracks month-to-month changes in financial circumstances and mental health (measured by GAD-7 for anxiety and PHQ-8 for depression) of panels recruited in the UK and France. The authors found expected overall socioeconomic gradients in anxiety and depression and associations between monthly fluctuations in financial situation and fluctuations in anxiety and depression. Crucially, and separately from income, increases in essential costs had an immediate impact on depression. While there are some costs that might increase due to ill-health – for example in relation to electricity if individuals require their home to be heated for longer due to having to remain at home more often – this impact is likely to be smaller overall than changes in income. Indeed, the Changing Cost of Living Study has tracked the same individuals during a period of energy price shocks and it is therefore likely that much of the increase in costs is due to exogenous, non-health-related factors.

In addition, there is a broad range of evidence from previous generations of research that socioeconomic inequalities have an impact on health. Indeed, a key lesson of the Whitehall II study of civil servants was that, in contrast to the received opinion that the most senior managers in organisations were subject to heighted ‘executive stress’, those lower down the chain had biomarkers indicative of greater stress and ill-health and, indeed, increased rates of actual ill-health, such as cardiovascular disease and premature mortality [21]. Russ and colleagues’ [71] more recent pooled meta-analysis of the relationship between chronic stress and mortality from 10 prospective cohort studies found that greater distress was associated with ‘increased risk of mortality from several major causes in a dose-response pattern’. Indeed, some of the authors of the present study have presented chronic biopsychosocial stress and resulting glucocorticoid resistance as a plausible biological pathway from insecure, inadequate non-committed income to health [70]. Further, if the causal relationship from income to health is not a significant one, it would be difficult to explain the substantially worse health outcomes in developing countries which often have younger, and therefore prospectively healthier, populations as well as the demonstrable benefits gleaned from economic interventions in those countries.

Given the objectives to provide a means of modelling health effects accurately based on income redistribution, we believe the within-between analyses provide the best estimate of the effect of income on health possible from observational data, with controls in place that likely err on the conservative. This does not mean that the analysis is without limitations, and the inability to assert causation without significant caveats is an important consideration in assessing the case for further research. Indeed, an assessment of this case using Value of Information (VoI) [72] methods is planned by the authorship team.

## Conclusion

Our analysis in this article using within-between models and UKHLS data with a number of specifications confirms previous findings, including some of the authors’ own with regard to mental health among 16- to 24-year-olds [3], of a positive association between average income and income changes on the one hand and mental and physical health on the other among adults aged 18 and over. Building on previous analysis of the relationship between income and mental health among adolescents and young adults [3], our findings here enable microsimulation modelling of the prospective national impacts of upstream economic interventions such as welfare policies for both mental and physical health and among the whole adult population. We have also shown that policies that redistribute more income to lower points in the distribution, such as Basic Income accompanied by progressive tax reform, are likely to result in greater health benefits than those that increase incomes to a smaller degree across the whole population, such as tax changes alone. Further work in an experimental context is needed to confirm these findings. However, quasi-experimental studies that focus on the effects of increased costs may also help to build the case for causation as these are less likely to be theoretically affected by ill-health.

## Supporting information

S1 Appendix Descriptive statistics, random effects logistic panel regressions (Model 2) tables and figures, and unlagged and three-year lagged comparisons for Table 2 . (DOCX)

S1 Fig Predicted effect of £100 per month increase in equivalised household net income on probability of MCS-12 score being ≤ 45.6 and PCS-12 being ≤ 50.0. Note: Specification 2 is omitted because the coefficients on the between-income variables are almost identical to Specification 1 (to within.001).
(TIF)
