# Supplementary material for: Examining the relationship between income and both mental and physical health among adults in the UK: Analysis of 12 waves (2009–2022) of Understanding Society
Source: PLoS One. 2025 Mar 6;20(3):e0316792. doi: 10.1371/journal.pone.0316792 (PMC11884696; doi:10.1371/journal.pone.0316792)
Supplement: S1 Appendix — (DOCX) [file pone.0316792.s001.docx]

**Examining the relationship between income and both mental and physical health among adults in the UK: Analysis of 12 waves (2009-2022) of Understanding Society**

**S1 Appendix**

## **Descriptive statistics**

**S1 Table. Sample size of unbalanced UKHLS panel and comparison with balanced panel**

| **Wave** | **Balanced panel** | **Unbalanced panel** | **Balanced panel as % of unbalanced** |
| --- | --- | --- | --- |
| **1 (2009-11)** | 6,649 | 46,935 | 14.2% |
| **2 (2010-12)** | 6,649 | 39,770 | 16.7% |
| **3 (2011-13)** | 6,649 | 40,489 | 16.4% |
| **4 (2012-14)** | 6,649 | 39,151 | 17.0% |
| **5 (2013-15)** | 6,649 | 37,032 | 18.0% |
| **6 (2014-16)** | 6,649 | 35,089 | 18.9% |
| **7 (2015-17)** | 6,649 | 36,834 | 18.1% |
| **8 (2016-18)** | 6,649 | 35,151 | 18.9% |
| **9 (2017-19)** | 6,649 | 32,118 | 20.7% |
| **10 (2018-20)** | 6,649 | 30,792 | 21.6% |
| **11 (2019-21)** | 6,649 | 29,223 | 22.8% |
| **12 (2020-22)** | 6,649 | 27,376 | 24.3% |
| **Total** | 79,788 | 429,960 | 18.6% |

**S2 Table. Descriptive statistics for Understanding Society sample aged 18 and over: unbalanced and balanced panels, means of variables**

|  | **Unbalanced panel** | **Balanced panel** |
| --- | --- | --- |
| **Number of individuals (N)** | 80,009 | 6,649 |
| **Number of observations (N x T)** | 429,960 | 79,788 |
| **Health:** |  |  |
| SF-12 Mental Component Summary (MCS-12) score | 49.50 | 50.47 |
| SF-12 Physical Component Summary (PCS-12) score | 49.07 | 50.18 |
| **Age** |  |  |
| 18-24 | 12.58% | 1.98% |
| 25-34 | 13.80% | 9.51% |
| 35-44 | 17.21% | 17.04% |
| 45-54 | 18.44% | 22.21% |
| 55-64 | 16.16% | 23.68% |
| 65-74 | 13.43% | 19.25% |
| 75+ | 8.38% | 6.33% |
| **Gender** |  |  |
| Male | 44.36% | 42.34% |
| Female | 55.64% | 57.66% |
| **Ethnicity*** |  |  |
| White British/English/Scottish/Welsh/Northern Irish | 80.00% | 90.16% |
| White (other)* | 4.66% | 3.25% |
| Mixed** | 1.71% | 1.22% |
| South Asian (Indian, Pakistani, Bangladeshi) | 7.47% | 2.61% |
| Chinese and any other Asian background | 1.36% | 0.86% |
| Caribbean, African and any other black background | 3.37% | 1.61% |
| Other | 0.68% | 0.29% |
| **Country of birth** |  |  |
| Born in the UK | 85.04% | 92.30% |
| Not born in the UK | 14.96% | 7.70% |
| **Nation within UK** |  |  |
| England | 78.22% | 86.09% |
| Scotland | 8.79% | 7.60% |
| Wales | 6.78% | 3.70% |
| Northern Ireland | 6.17% | 2.60% |
| **Marital status** |  |  |
| Single, never married | 22.18% | 11.60% |
| Cohabiting | 11.00% | 9.46% |
| Married/in a civil partnership | 52.72% | 63.96% |
| Divorced | 6.47% | 8.16% |
| Widowed | 5.73% | 5.10% |
| Other | 1.89% | 1.70% |
| **Occupational classification (NS-SEC) based on current job** |  |  |
| Managerial and professional | 24.27% | 30.50% |
| Intermediate | 7.85% | 9.22% |
| Small employers and self-employed | 5.40% | 5.31% |
| Lower supervisory and technical | 3.98% | 3.39% |
| Semi-routine and routine | 15.23% | 11.95% |
| Not employed | 42.12% | 38.78% |
| Missing | 1.16% | 0.86% |
| **Labour market status** |  |  |
| Employed | 55.59% | 60.17% |
| Unemployed | 4.59% | 2.14% |
| Family care | 5.03% | 3.74% |
| Full-time student | 6.33% | 0.90% |
| Long term sick or disabled | 3.27% | 2.12% |
| Retired | 23.64% | 29.70% |
| Other | 1.54% | 1.22% |
| **Education: highest qualification** |  |  |
| University degree | 25.40% | 34.46% |
| Other higher education (e.g. professional qualifications) | 12.00% | 15.34% |
| A level | 21.03% | 17.69% |
| GCSE/ O Level | 20.38% | 18.27% |
| Other qualifications | 8.91% | 8.18% |
| No qualifications | 12.28% | 6.06% |
| **Household level variables:** |  |  |
| **Number of household observations** | 260,280 | 61,948 |
| **Household income:** |  |  |
| Net household monthly income before housing costs (unequivalised) | £3,645.03 | £3,993.02 |
| Net household monthly income before housing costs (equivalised) | £2,169.26 | £2,411.80 |
| **Housing costs** | £328.74 | £266.97 |
| **Household tenure:** |  |  |
| Own (outright or with mortgage) | 69.69% | 81.44% |
| Renting: local authority or housing association | 13.14% | 8.61% |
| Renting: private landlord | 17.17% | 9.95% |
| **Household composition:** |  |  |
| Average number of adults using OECD definition (age 14+) | 2.08 | 2.08 |
| Average number of children using OECD definition (age 0-13) | 0.46 | 0.38 |

* Uses ‘racel’ variable

** Includes: Irish, gypsy or Irish traveller, and any other white background

*** Includes: white and black Caribbean, white and black African, white and Asian, and any other mixed background

**S3 Table. Number and percentage of respondents with an SF-12 MCS of ≤45.6 indicating clinical depressive disorder and with an SF-12 PCS score of ≤50.0 indicating a physical health problem by wave of interview: unbalanced panel**

|  | **SF-12 MCS** | | **SF-12 PCS** | | **Total number** |
| --- | --- | --- | --- | --- | --- |
|  | **Clinically significant score of ≤45.6 indicating depressive disorder** | **Score of ≥45.7** | **Clinically significant score of ≤50 indicating physical health problem** | **Score of ≥50** |  |
| **Wave 1 (2009-10)** | 24.20% | 75.80% | 35.82% | 64.18% | 46,965 |
| **Wave 2 (2010-11)** | 26.40% | 73.60% | 36.11% | 63.89% | 39,770 |
| **Wave 3 (2011-12)** | 28.95% | 71.05% | 37.80% | 62.20% | 40,489 |
| **Wave 4 (2012-13)** | 28.61% | 71.39% | 37.34% | 62.66% | 39,151 |
| **Wave 5 (2013-14)** | 29.64% | 70.36% | 38.44% | 61.56% | 37,032 |
| **Wave 6 (2014-15)** | 27.41% | 72.59% | 38.07% | 61.93% | 35,089 |
| **Wave 7 (2015-16)** | 31.35% | 68.65% | 36.78% | 63.22% | 36,834 |
| **Wave 8 (2016-17)** | 32.12% | 67.88% | 37.68% | 62.32% | 35,151 |
| **Wave 9 (2017-18)** | 32.64% | 67.36% | 37.70% | 62.30% | 32,118 |
| **Wave 10 (2018-19)** | 35.12% | 64.88% | 39.43% | 60.57% | 30,792 |
| **Wave 11 (2019-20)** | 36.25% | 63.75% | 38.44% | 61.56% | 29,223 |
| **Wave 12 (2020-21)** | 36.46% | 63.54% | 37.82% | 62.18% | 27,376 |
| **Total** | 30.26% | 69.74% | 37.52% | 62.48% | 429,960 |

**S4 Table. Number and percentage of respondents with an SF-12 MCS of ≤45.6 indicating clinical depressive disorder and with an SF-12 PCS score of ≤50.0 indicating a physical health problem by wave of interview: balanced panel**

|  | **SF-12 MCS** | | **SF-12 PCS** | | **Total number** |
| --- | --- | --- | --- | --- | --- |
|  | **Clinically significant score of ≤45.6 indicating depressive disorder** | **Score of ≥45.7** | **Clinically significant score of ≤50 indicating physical health problem** | **Score of ≥50** |  |
| **Wave 1 (2009-10)** | 18.29% | 81.71% | 28.56% | 71.44% | 6,649 |
| **Wave 2 (2010-11)** | 21.88% | 78.12% | 28.56% | 71.44% | 6,649 |
| **Wave 3 (2011-12)** | 24.03% | 75.97% | 31.46% | 68.54% | 6,649 |
| **Wave 4 (2012-13)** | 22.97% | 77.03% | 30.77% | 69.23% | 6,649 |
| **Wave 5 (2013-14)** | 25.18% | 74.82% | 32.88% | 67.12% | 6,649 |
| **Wave 6 (2014-15)** | 22.50% | 77.50% | 34.37% | 65.63% | 6,649 |
| **Wave 7 (2015-16)** | 25.09% | 74.91% | 33.76% | 66.24% | 6,649 |
| **Wave 8 (2016-17)** | 26.82% | 73.18% | 35.81% | 64.19% | 6,649 |
| **Wave 9 (2017-18)** | 26.77% | 73.23% | 37.03% | 62.97% | 6,649 |
| **Wave 10 (2018-19)** | 28.94% | 71.06% | 39.80% | 60.20% | 6,649 |
| **Wave 11 (2019-20)** | 30.52% | 69.48% | 40.20% | 59.80% | 6,649 |
| **Wave 12 (2020-21)** | 31.39% | 68.61% | 40.47% | 59.53% | 6,649 |
| **Total** | 30.26% | 74.64% | 34.47% | 65.53% | 79,788 |

## **Random effects logistic panel regressions (Model 2): Probability of PCS-12 and MCS-12 score below critical thresholds**

S5-S8 Tables present the results for the random effects logistic panel regressions (Model 2). As with Tables 3, 4 and 5 in the main manuscript, the tables below show the within-income and between-income coefficients only. The unbalanced panel marginal effects (with standard errors) are shown in the two left-hand columns, with the balanced panel marginal effects shown in the right-hand columns.

#### Specification 1: log-linear

S5 Table shows the results from Model 2, Specification 1. For the unbalanced panel, the marginal effects are negative and significant at the 1% level. For the probability of MCS-12 being equal to or below the threshold level of 45.6, the between-income coefficient is around -0.045, and for the probability of PCS being equal to or below the threshold level of 50, the between income coefficient is around -0.04. The equivalent coefficients for the balanced panel models are also significant, and larger in absolute terms (around -0.07 for the balanced MCS-12 model and -0.06 for the balanced PCS model).

As with the continuous models in Tables 1 and 2, the within-income coefficients are mostly either insignificant or of borderline significance (for example, the within-income coefficient for the unbalanced MCS-12 model is significant at the 10% level). Only for the PCS balanced panel model is the within-income effect significant at the 5% level (and relatively small, at around +0.008).

**S5 Table. Results from Model 2, Specification 1 (log-linear)**

|  | **Unbalanced panel**  **Marginal effects (SE):** | | **Balanced panel**  **Marginal effects (SE):** | |
| --- | --- | --- | --- | --- |
| **Log income:** | **Pr (MCS-12 ≤45.6)** | **Pr (PCS-12 ≤50.0)** | **Pr (MCS-12 ≤45.6)** | **Pr (PCS-12 ≤50.0)** |
| Between-income | -0.0452*** (0.0025) | -0.0399*** (0.0023) | -0.0689*** (0.0067) | -0.0602*** (0.00655) |
| Within-income | -0.0035* (0.0018) | 0.0025 (0.0017) | -0.0002 (0.0037) | 0.0077** (0.0039) |
| Observations | 315,093 | 315,093 | 73,139 | 73,139 |
| **Individuals** | 58,729 | 58,729 | 6,649 | 6,649 |
| **Log likelihood** | -149142.45 | -133477.87 | -30977.006 | -30297.282 |

∗ p < 0.10, ∗∗ p < 0.05, ∗∗∗ p < 0.01

**Specification 2: within-between interactions**

S6 Table presents results from Specification 2, which contains a within-between interaction term for incomes. The between-income coefficients are identical to Specification 1. None of the within-income or within-between interaction terms are significant except for in the balanced panel MCS-12 model, where the coefficients are significant at the 10% level.

**S6 Table. Results from Model 2, Specification 2 (within-between interaction)**

|  | **Unbalanced panel**  **Marginal effects (SE):** | | **Balanced panel**  **Marginal effects (SE):** | |
| --- | --- | --- | --- | --- |
| **Log income:** | **Pr (MCS-12 ≤45.6)** | **Pr (PCS-12 ≤50.0)** | **Pr (MCS-12 ≤45.6)** | **Pr (PCS-12 ≤50.0)** |
| Between-income | -0.0452*** (0.0025) | -0.0399*** (0.0023) | -0.0689*** (0.0067) | -0.0602*** (0.0065) |
| Within-income | 0.0176 (0.0169) | 0.0166 (0.0166) | 0.0859* (0.0514) | -0.0171 (0.0533) |
| Within-between interaction | -0.0030 (0.0024) | -0.0020 (0.0023) | -0.0117* (0.0069) | 0.0033 (0.0072) |
| **Observations** | 315,093 | 315,093 | 73,139 | 73,139 |
| **Individuals** | 58,729 | 58,729 | 6,649 | 6,649 |
| **Log likelihood** | -149141.66 | -133477.5 | -30975.588 | -30297.174 |

∗ p < 0.10, ∗∗ p < 0.05, ∗∗∗ p < 0.01

**Specification 3: log-level interactions**

S7 Table presents results from Specification 3, which includes an interaction term between log incomes and income levels. For between-incomes, the log-level interaction terms are significant at the 1% level for the unbalanced panel but are insignificant for the balanced panel. In the unbalanced panel, the log-level coefficients (multiplied by 100,000) are around -0.035 for the MCS-12 model and -0.03 for the PCS-12 model. The log income coefficients in each of the models in S7 Table is slightly smaller (in absolute terms) than the coefficients in Specifications 1 and 2.

**S7 Table. Results from Model 2, Specification 3 log-level interactions)**

|  | **Unbalanced panel**  **Marginal effects (SE):** | | | **Balanced panel**  **Marginal effects (SE):** | | |
| --- | --- | --- | --- | --- | --- | --- |
| **Log income:** | **Pr (MCS-12 ≤45.6)** | **Pr (PCS-12 ≤50.0)** | **Pr (MCS-12 ≤45.6)** | | **Pr (PCS-12 ≤50.0)** |  |
| **Between-income:** |  |  |  | |  |  |
| Log | -0.0382*** (0.0034) | -0.0338*** (0.0032) | -0.0632*** (0.0115) | | -0.0548*** (0.0111) |  |
| Log x levels (x100000) | -0.0347***(0.0123) | -0.0297*** (0.0109) | -0.0249 (0.0416) | | -0.0237 (0.0395) |  |
| **Within-Income:** |  |  |  | |  |  |
| Log | -0.0032*(0.0019) | 0.0024 (0.0018) | 0.0000 (0.0037) | | 0.0076* (0.0039) |  |
| Log x levels (x100000) | -0.0260 (0.0224) | 0.0061 (0.0086) | -0.0296 (0.0437) | | 0.0057 (0.0163) |  |
| **Observations** | 315,093 | 315,093 | 73,139 | | 73,139 |  |
| **Individuals** | 58,729 | 58,729 | 6,649 | | 6,649 |  |
| **Log likelihood** | -149135.52 | -133473.82 | -30976.306 | | -30297.063 |  |

∗ p < 0.10, ∗∗ p < 0.05, ∗∗∗ p < 0.01

**Specification 4: quintile interactions**

Finally in this section, S8 Table presents the results from Specification 4, where the between-income and within-income effects are interacted with income quintile dummy variables. For the MCS-12, the results for the unbalanced panel show that the between-income quintile dummies are positive and significant compared to the base quintile (quintile 5, i.e. the top income quintile). When combined with the negative coefficient on (uninteracted) log income, this suggests a slightly weaker negative relationship between increases log income and the probability of MCS-12 being below the threshold of 45.6 for people in households in lower income quintiles compared to the top quintile. The results for the unbalanced PCS-12 panel also show positive coefficients for the between-income quintile dummies 1 to 4 and a (larger) overall negative coefficient for the uninteracted log income term.

For the balanced panel, the between-income quintile dummies are insignificant in the MCS-12 model, but significant (and positive) in the PCS-12 model. The within-income coefficients in these models are mostly insignificant with the exception of the quintile 4 interaction term which is positive and significant for the MCS-12 and PCS-12 unbalanced models and the MCS-12 balanced model. The dummies for quintiles 2 and 3 in the MCS-12 unbalanced model are also significant (and positive).

**S8 Table. Results from Model 2, Specification 4 (quintile interactions)**

|  | **Unbalanced panel**  **Marginal effects (SE):** | | **Balanced panel**  **Marginal effects (SE):** | |
| --- | --- | --- | --- | --- |
| **Log income:** | **Pr (MCS-12 ≤45.6)** | **Pr (PCS-12 ≤50.0)** | **Pr (MCS-12 ≤45.6)** | **Pr (PCS-12 ≤50.0)** |
| **Between-income:** |  |  |  |  |
| Log | -0.0260*** (0.0038) | -0.0305*** (0.0035) | -0.0516*** (0.0095) | -0.0405*** (0.0095) |
| Log x quintile 1 | 0.0037*** (0.0006) | 0.0021*** (0.0007) | 0.0025* (0.0015) | 0.0037*** (0.0015) |
| Log x quintile 2 | 0.0037*** (0.0005) | 0.0020*** (0.0005) | 0.0028** (0.0012) | 0.0041*** (0.0013) |
| Log x quintile 3 | 0.0026*** (0.0005) | 0.0013*** (0.0004) | 0.0012 (0.0009) | 0.0027*** (0.0010) |
| Log x quintile 4 | 0.0014*** (0.0004) | 0.0008** (0.0004) | 0.0002 (0.0007) | 0.0017** (0.0007) |
| **Within-Income:** |  |  |  |  |
| Log | 0.0049 (0.0051) | 0.0011 (0.0047) | -0.0003 (0.0099) | 0.0205** (0.0098) |
| Log x quintile 1 | -0.0085 (0.0058) | 0.0070 (0.0054) | 0.0026 (0.0116) | -0.0068 (0.0119) |
| Log x quintile 2 | 0.0205** (0.0089) | 0.0096 (0.0084) | 0.0185 (0.0192) | 0.0120 (0.0197) |
| Log x quintile 3 | 0.0292*** (0.0087) | 0.0112 (0.0082) | 0.0325 (0.0181) | 0.0005 (0.0185) |
| Log x quintile 4 | 0.0320*** (0.0085) | 0.0245*** (0.0080) | 0.0440*** (0.0169) | 0.0159 (0.0172) |
| **Observations** | 315,093 | 315,093 | 73,139 | 73,139 |
| **Individuals** | 58,729 | 58,729 | 6,649 | 6,649 |
| **Log likelihood** | -149111.12 | -133464.0 | -30970.857 | -30291.33 |

∗ p < 0.10, ∗∗ p < 0.05, ∗∗∗ p < 0.01

### Visualising coefficients on between-incomes for Model 2, Specifications 1, 3 and 4

As with Model 1 above, the combination of income coefficients for Specification 2, 3 and 4, and the fact that log income is used for the main income measures, makes it harder to interpret the size of the predicted income coefficient in the regressions for Model 2. Fig S1 shows the predicted effects of a £100 per month increase in equivalised household net income on the probability of MCS-12 score being equal to or below the threshold level of 45.6 (Panel A) and PCS-12 score being equal to or below 50.0. Panel (A) shows that for Specifications 1 and 3, in quintile 1 (the left-hand point of the diagram, corresponding to average household income of just under £1,000 per month) an increase of £100 per month reduces the probability of being below the MCS-12 threshold level by around 1 percentage point (or 0.01). Equivalently, an increase of £100 per month *increases* the probability of being above the MCS-12 threshold level by 1 percentage point. The marginal effects are progressively smaller in absolute terms at higher points the income distribution. At quintile 3 the marginal effect is around 0.6 percentage points for Specifications 1 and 3, and at quintile 5 the marginal effect is between 0.3 and 0.4 percentage points. For Specification 4, the general shape of the marginal effects by quintile is similar to the other specifications but the effects are smaller in absolute terms, ranging from around -0.8 percentage points for quintile 1 to -0.2 percentage points for quintile 5. Panel (B) shows similar overall patterns to Panel (B). For Specifications 1 and 3, a £100 increase in household income in the lowest income quintile is associated with a reduction of around 0.9 percentage points in the probability of PCS-12 score being below the threshold level. For the middle income quintile the marginal effect is just over -0.5 percentage points, and for the top income quintile it is -0.3 percentage points. Once again, Specification 4 exhibits the same general pattern of marginal effects by income quintile (with the effects getting smaller in absolute terms as income rises) but the absolute size of the effects is smaller.

Overall, Fig S1 shows that increases in income have a positive relationship to MCS-12 and PCS-12 scores. While Fig 3 (from Model 1) show that increased income is associated with higher MCS-12 and PCS-12 on the continuous scale, Fig S1 (from Model 2) show that increased income is associated with a lower probability of MCS-12 or PCS-12 being below critical threshold levels. Thus, the two sets of results are complementary.

**
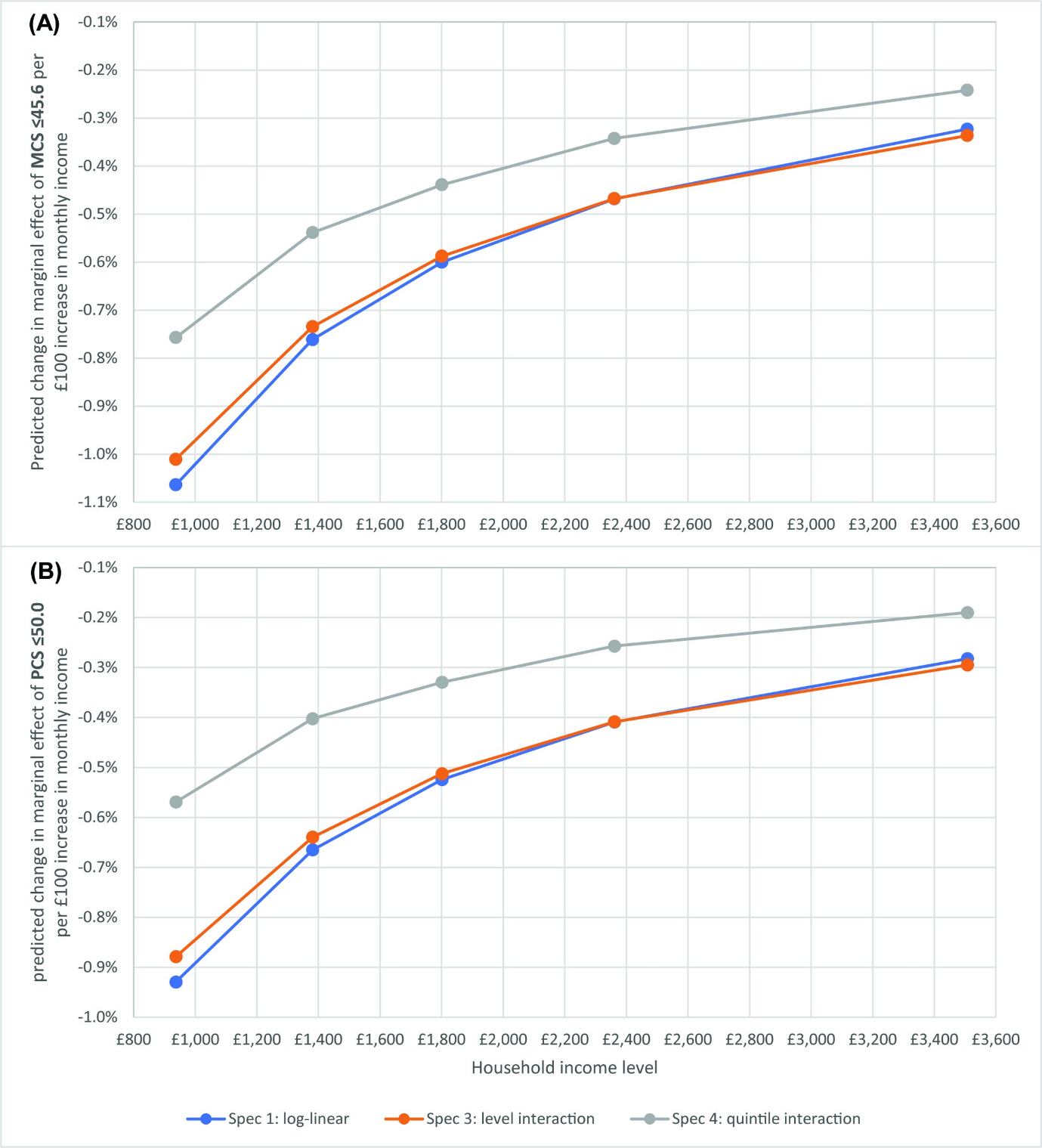
**

**S1 Fig. Predicted effect of £100 per month increase in equivalised household net income on probability of MCS-12 score being ≤45.6 and PCS-12 being ≤50.0**

Note: Specification 2 is omitted because the coefficients on the between-income variables are almost identical to Specification 1 (to within .001).

## **Unlagged and three-year lagged comparisons for Table 2**

**S9 Table. MCS-12 specification 1 balanced panel linear regression - unlagged and three-year lag**

|  | **Coefficient** | **SE** | **p value** |
| --- | --- | --- | --- |
| **Income measures:** |  |  |  |
| Average log BHC income ('between individual' measure) | 0.676273 | 0.085262 | 0 |
| Unlagged change in log BHC income ('within individual' measure) | -0.030687 | 0.080302 | 0.702 |
| Three-year lag change in log BHC income ('within individual' measure) | -0.041450 | 0.080176 | 0.605 |
| **Gender:** |  |  |  |
| Female | -0.744222 | 0.063662 | 0 |
| Male | REF | REF | REF |
| **Ethnicity:** |  |  |  |
| Mixed | -0.386612 | 0.281937 | 0.17 |
| Asian | -0.642615 | 0.185657 | 0.001 |
| Black | 0.969316 | 0.257345 | 0 |
| Other | -1.003919 | 0.573913 | 0.08 |
| White | REF | REF | REF |
| **Place of birth:** |  |  |  |
| UK | -0.372808 | 0.127947 | 0.004 |
| Outside UK | REF | REF | REF |
| **Limiting long-standing illness or disability status** |  |  |  |
| Limiting long-standing illness or disability | -1.238356 | 0.066646 | 0 |
| No limiting long-standing illness or disability | REF | REF | REF |
| **Marital status** |  |  |  |
| Married or in civil partnership | 0.564518 | 0.091523 | 0 |
| Divorced or separated | 0.101354 | 0.119702 | 0.397 |
| Widowed | 0.532061 | 0.160790 | 0.001 |
| Single never married | REF | REF | REF |
| **Age group** |  |  |  |
| 18-24 | REF | REF | REF |
| 25-34 | -1.135050 | 0.306069 | 0 |
| 35-44 | -0.918463 | 0.300830 | 0.002 |
| 45-54 | -0.501556 | 0.299616 | 0.094 |
| 55-64 | 0.252082 | 0.302623 | 0.405 |
| 65-74 | 0.719812 | 0.319182 | 0.024 |
| 75+ | 0.976037 | 0.337253 | 0.004 |
| **Highest in household qualification** |  |  |  |
| Degree | -0.464205 | 0.147367 | 0.002 |
| Other (non-degree) higher education | -0.324692 | 0.152801 | 0.034 |
| A level or equivalent | -0.230758 | 0.149856 | 0.124 |
| GCSE or equivalent | -0.056153 | 0.147607 | 0.704 |
| Other | -0.121025 | 0.164722 | 0.463 |
| No qualification | REF | REF | REF |
| **Economic status** |  |  |  |
| Employee | 3.042142 | 0.182498 | 0 |
| Self-employed | 3.287106 | 0.207656 | 0 |
| Looking after family | 2.701801 | 0.234332 | 0 |
| Unemployed | 1.392162 | 0.281112 | 0 |
| Retired | 3.590015 | 0.203511 | 0 |
| Working-age inactive | REF | REF | REF |
| **Urban or rural** |  |  |  |
| Rural | 0.031133 | 0.071640 | 0.664 |
| Urban | REF | REF | REF |
| **Nation or region** |  |  |  |
| North East | REF | REF | REF |
| North West | 0.126723 | 0.164985 | 0.442 |
| Yorkshire and the Humber | 0.343153 | 0.174584 | 0.049 |
| East Midlands | 0.384398 | 0.171632 | 0.025 |
| West Midlands | -0.018501 | 0.170683 | 0.914 |
| East | 0.075398 | 0.166225 | 0.65 |
| London | -0.038253 | 0.182298 | 0.834 |
| South East | 0.091885 | 0.157596 | 0.56 |
| South West | 0.119993 | 0.164340 | 0.465 |
| Wales | -0.151395 | 0.209205 | 0.469 |
| Scotland | 0.211313 | 0.176096 | 0.23 |
| Northern Ireland | -0.013508 | 0.233256 | 0.954 |
| **Housing tenure** |  |  |  |
| Own home | 0.537231 | 0.121899 | 0 |
| Social renter (local authority/housing association) | -0.553534 | 0.155141 | 0 |
| Private renter | REF | REF | REF |
| **Constant** | 14.076630 | 0.731884 | 0 |
| **L1. (First lag)** | 0.563484 | 0.003444 | 0 |

n=6,649. Observations=59,841. R-squared: within=0.0050; between=0.9188; overall=0.4054

**S10 Table. PCS-12 specification 1 balanced panel linear regression - unlagged and three-year lag**

|  | **Coefficient** | **SE** | **p value** |
| --- | --- | --- | --- |
| **Income measures** |  |  |  |
| Average log BHC income ('between individual' measure) | 0.507459 | 0.073741 | 0 |
| Unlagged change in log BHC income ('within individual' measure) | -0.040220 | 0.069430 | 0.562 |
| Three-year lag change in log BHC income ('within individual' measure) | -0.050626 | 0.069317 | 0.465 |
| **Gender** |  |  |  |
| Female | -0.178966 | 0.054892 | 0.001 |
| Male | REF | REF | REF |
| **Ethnicity** |  |  |  |
| Mixed | -0.361283 | 0.243760 | 0.138 |
| Asian | -0.593248 | 0.160573 | 0 |
| Black | 0.510143 | 0.222466 | 0.022 |
| Other | -0.323680 | 0.496108 | 0.514 |
| White | REF | REF | REF |
| **Place of birth** |  |  |  |
| UK | 0.199125 | 0.110605 | 0.072 |
| Outside UK | REF | REF | REF |
| **Limiting long-standing illness or disability status** |  |  |  |
| Limiting long-standing illness or disability | -3.598565 | 0.061895 | 0 |
| No limiting long-standing illness or disability | REF | REF | REF |
| **Marital status** |  |  |  |
| Married or in civil partnership | -0.133889 | 0.079063 | 0.09 |
| Divorced or separated | -0.229121 | 0.103496 | 0.027 |
| Widowed | -0.336442 | 0.139033 | 0.016 |
| Single never married | REF | REF | REF |
| **Age group** |  |  |  |
| 18-24 | REF | REF | REF |
| 25-34 | -0.889663 | 0.264586 | 0.001 |
| 35-44 | -1.339698 | 0.260176 | 0 |
| 45-54 | -1.715553 | 0.259335 | 0 |
| 55-64 | -2.146332 | 0.262137 | 0 |
| 65-74 | -2.416449 | 0.276461 | 0 |
| 75+ | -3.342392 | 0.292390 | 0 |
| **Highest in household qualification** |  |  |  |
| Degree | 1.296758 | 0.127804 | 0 |
| Other (non-degree) higher education | 0.889697 | 0.132291 | 0 |
| A level or equivalent | 0.947090 | 0.129781 | 0 |
| GCSE or equivalent | 0.600706 | 0.127713 | 0 |
| Other | 0.431945 | 0.142451 | 0.002 |
| No qualification | REF | REF | REF |
| **Economic status** |  |  |  |
| Employee | 3.626970 | 0.159617 | 0 |
| Self-employed | 3.750393 | 0.181064 | 0 |
| Looking after family | 3.340422 | 0.203613 | 0 |
| Unemployed | 3.314068 | 0.244354 | 0 |
| Retired | 3.141764 | 0.176632 | 0 |
| Working-age inactive | REF | REF | REF |
| **Urban or rural** |  |  |  |
| Rural | 0.114407 | 0.061948 | 0.065 |
| Urban | REF | REF | REF |
| **Nation or region** |  |  |  |
| North East | REF | REF | REF |
| North West | -0.141546 | 0.142648 | 0.321 |
| Yorkshire and the Humber | 0.036773 | 0.150940 | 0.808 |
| East Midlands | 0.211451 | 0.148392 | 0.154 |
| West Midlands | -0.086423 | 0.147564 | 0.558 |
| East | 0.081852 | 0.143720 | 0.569 |
| London | 0.045700 | 0.157610 | 0.772 |
| South East | 0.188120 | 0.136262 | 0.167 |
| South West | 0.196785 | 0.142093 | 0.166 |
| Wales | 0.082868 | 0.180874 | 0.647 |
| Scotland | 0.041600 | 0.152250 | 0.785 |
| Northern Ireland | -0.387147 | 0.201708 | 0.055 |
| **Housing tenure** |  |  |  |
| Own home | 0.408388 | 0.105329 | 0 |
| Social renter (local authority/housing association) | -0.440442 | 0.134226 | 0.001 |
| Private renter | REF | REF | REF |
| **Constant** | 13.191850 | 0.633777 | 0 |
| **L1. (First lag)** | 0.630156 | 0.003114 | 0 |

n=6,649. Observations=59,841. R-squared: within=0.0375; between=0.9520; overall=0.6115
