# Supplementary figures and images for: Examining the relationship between income and both mental and physical health among adults in the UK: Analysis of 12 waves (2009–2022) of Understanding Society
Source: PLoS One. 2025 Mar 6;20(3):e0316792. doi: 10.1371/journal.pone.0316792 (PMC11884696; doi:10.1371/journal.pone.0316792)

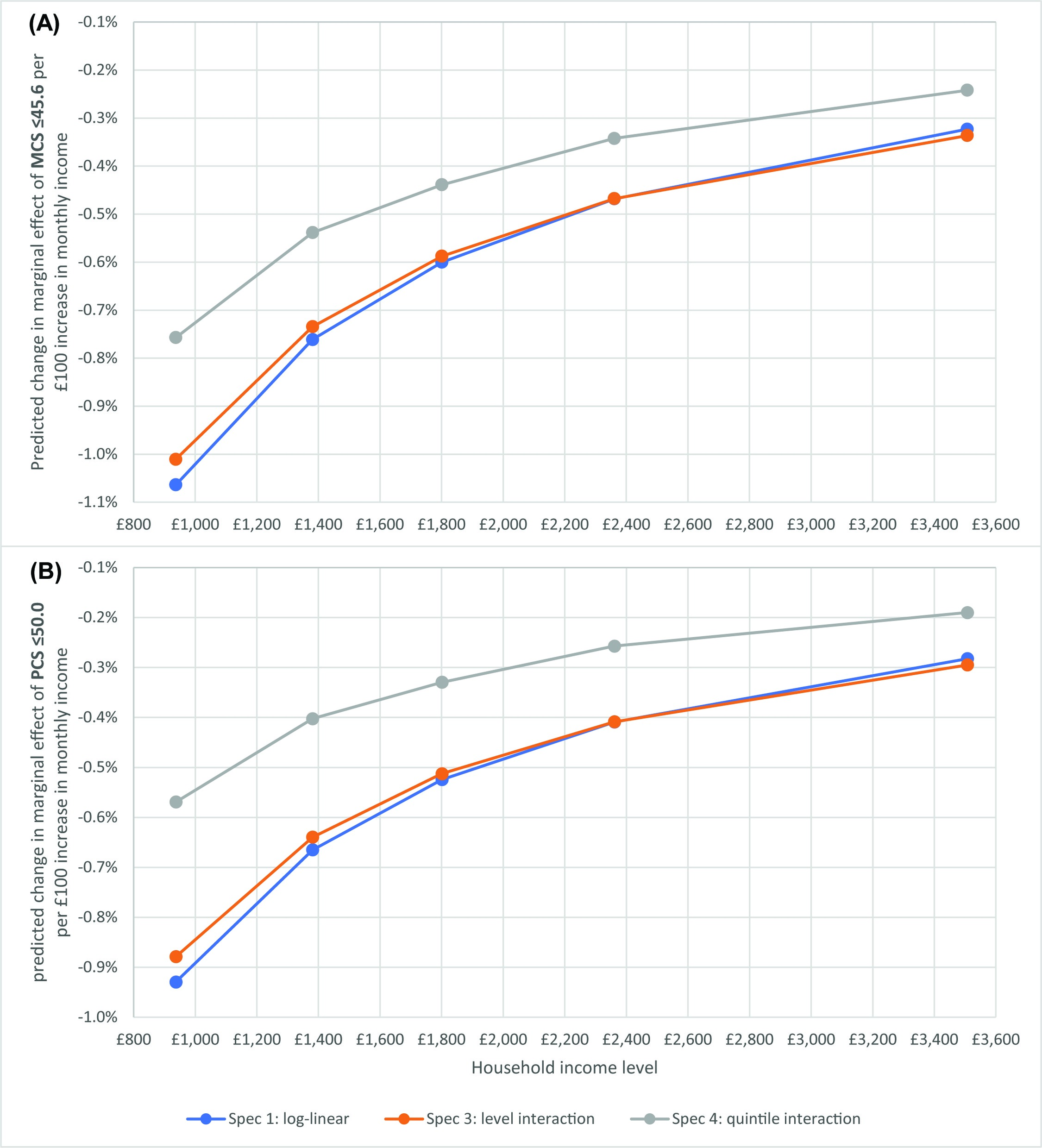

Supplement: S1 Fig — Note: Specification 2 is omitted because the coefficients on the between-income variables are almost identical to Specification 1 (to within.001). (TIF) [file pone.0316792.s002.tif]
